# Ferrocene-Containing Impiridone (ONC201) Hybrids: Synthesis, DFT Modelling, In Vitro Evaluation, and Structure–Activity Relationships

**DOI:** 10.3390/molecules23092248

**Published:** 2018-09-03

**Authors:** Péter Bárány, Rita Szabó Oláh, Imre Kovács, Tamás Czuczi, Csenge Lilla Szabó, Angéla Takács, Eszter Lajkó, Orsolya Láng, László Kőhidai, Gitta Schlosser, Szilvia Bősze, Gábor Mező, Ferenc Hudecz, Antal Csámpai

**Affiliations:** 1 Institute of Chemistry, Eötvös Loránd University (ELTE), Budapest, H-1117 Budapest, Hungary; peterbarany@caesar.elte.hu (P.B.); kimre950518@gmail.com (I.K.); czuczi.tamas@gmail.com (T.C.); szabo.csenge44@gmail.com (C.L.S.); gitta.schlosser@gmail.com (G.S.); fhudecz@caesar.elte.hu (F.H.); 2 MTA-ELTE Research Group of Peptide Chemistry, Budapest Pázmány P. sétány 1/A, H-1117 Budapest, Hungary; rita.olah.szabo@gmail.com (R.S.O.); szilvia.bosze@gmail.com (S.B.); gmezo@caesar.elte.hu (G.M.); 3 Department of Genetics, Cell and Immunobiology, Semmelweis University, Nagyvárad tér 4, H-1089 Budapest, Hungary; angela.takacs1@gmail.com (A.T.); lajesz@gmail.com (E.L.); langorsi@gmail.com (O.L.); kohlasz2@gmail.com (L.K.)

**Keywords:** ferrocene, organic synthesis, NMR spectroscopy, DFT calculations, bioorganometallic chemistry, cytotoxic activity, structure–activity relationships

## Abstract

Inspired by the well-established clinical evidence about the interplay between apoptotic TRAIL (tumour necrosis factor-related apoptosis-inducing ligand) mechanism and reactive oxygen species (ROS)-mediated oxidative stress, a set of novel ONC201 hybrids containing the impiridone core and one or two differently positioned ferrocenylalkyl groups were synthesised in our present work. These two types of residues have been implicated in the aforementioned mechanisms associated with cytotoxic activity. A straightforward, primary amine-based synthetic approach was used allowing the introduction of a variety of *N*-substituents into the two opposite regions of the heterocyclic skeleton. Reference model compounds with benzyl and halogenated benzyl groups were also synthesised and tested. The in vitro assays of the novel impiridones on five malignant cell lines disclosed characteristic structure-activity relationship (SAR) featuring significant substituent-dependent activity and cell-selectivity. A possible contribution of ROS-mechanism to the cytotoxicity of the novel metallocenes was suggested by density functional theory (DFT)studies on simplified models. Accordingly, unlike the *mono*-ferrocenylalkyl-substituted products, the compounds containing two ferrocenylalkyl substituents in the opposite regions of the impiridone core display a much more pronounced long-term cytotoxic effect against A-2058 cell line than do the organic impiridones including ONC201 and ONC212. Furthermore, the prepared *bis*-metallocene derivatives also present substantial activity against COLO-205- and EBC-1 cell lines.

## 1. Introduction

**Figure 1:**
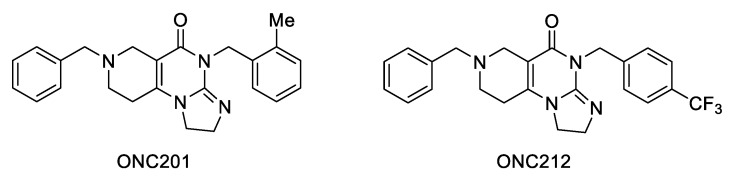
Representative impiridones as reference compounds.

A large diversity of tumours is among the most dreadful diseases of high mortality with poor prognosis regarding survival rate. Chemotherapy is generally considered as one of the essential tools for the treatment of malignancies. However, cisplatin, one of the most commonly used chemotherapeutics [1,2,3] with metal capable of covalently binding to DNA [4,5,6], gives rise to severe side-effects [7,8]. Although during the last decades important advances have been made leading to alternative therapeutic agents with remarkable structural diversity, the wide range of side effects remains one of the main problems in clinical therapy. Thus, to overcome toxic limitations and to broaden the scope of treatable malignancies there is a constant need for the development of further drug candidates with enhanced activity, selectivity, and bioavailability. Besides clinically approved classical metal-complexes (e.g., carboplatin and oxaliplatin) [9] and emblematic organic compounds (e.g., daunomycin, doxorubicin [10], vinblastine, and vincristine [11]), organometallics have also emerged as potential anticancer agents. Among organometallics, due to their nontoxic character and chemical stability, ferrocene derivatives with diverse molecular architectures and tunable redox properties are of pronounced importance, as supported by the reviews published in the last decade [12,13,14,15,16] indicating the rapidly growing interest towards bioorganometallic chemistry. It is of note that the ferrocene-containing analogues of the nonsteroidal selective estrogen receptor modulator 4-hydroxytamoxifen (Afimoxifene) [17], displaying strong cytotoxic and cytostatic effects on hormone-independent MDA-MB-231 breast tumour cells, are the most promising representatives of bioactive organometallics, under preclinical studies with established mechanisms of action [14]. In general, Fenton pathway-based redox chemistry of a pending ferrocene moiety in a potential therapeutic agent may play a key role in mitochondrial generation of reactive oxygen species (ROS), such as nitric oxide, superoxide anion, and other forms of free radicals [18,19,20] that have been shown to be involved in biological regulatory processes leading to programmed cell death (apoptosis) [21]. On the other hand, there is a growing interest in potential therapeutics with a capability to activate proapoptotic protein tumor necrosis factor-related apoptosis-inducing ligand (TRAIL) and its receptors. These molecules are able to mediate multiple intracellular signals finally triggering apoptosis in cancer cells leaving normal cells almost unaffected and thus, have a wide therapeutic index [22]. At this stage, it must be pointed out that convincing preclinical evidences have been disclosed about the interplay between TRAIL and redox signalling pathways implicated in cancer [23]. Recently, ONC201 (Figure 1) has emerged as a highly promising first-in-class small-molecule TRAIL inducer with a wide therapeutic index [24,25,26,27]. From the aspect of its mechanism of action, the following findings are worth pointing out. Kline et al. disclosed that ONC201 triggered dual inhibition of AKT and extracellular signal–regulated kinase (ERK) pathways in a number of malignant cell lines (e.g., HCT-116, HEPG-2, MCF-7, and MDA-MB-468) and demonstrated that, besides apoptosis measured by sub-G1 fraction and caspase activation, ONC201 also induced cell cycle arrest in the cell lines which were tested as early as 24 h after treatment [24]. By means of bromodeoxyuridine (BrdU) labelling experiments the authors also confirmed that the proliferation of the cells was inhibited by ONC201 and, as a response to the treatment with this impiridone, the early cell cycle arrest caused a significant decrease in a number of viable cells within 48 h, even including those (e.g., A-549 and SNV-449) that did not undergo apoptosis [24]. Preclinical studies have demonstrated its potency as an exceptionally promising apoptotic anticancer agent having pronounced activity against a large variety of cancer cell lines (including e.g., PANC-1, HCT116, MDA-MB-23, U87, HFF, MRC5, and WI-38) [28,29,30,31]. Moreover, in phase II clinical trials this compound has proved to be beneficial in the treatment of patients with a wide range of advanced malignances [32]. An intense search for analogues identified a trifluoromethylated derivative named as ONC212 (Figure 1) as a more potent impiridone producing enhanced activity at nanomolar concentrations against a number of different malignant cell lines, solid tumours, and hematological malignancies [33]. It is also of pronounced importance that ONC212 showed improved preclinical efficacy on pancreatic cancer, melanoma, and hepatocellular carcinoma in a few in vivo models including ONC201-resistant tumours, e.g., PANC-1 and Capan-2 human pancreatic cancer xenograft models [34].

## 2. Results and Discussion

Prompted by the exceptional success of certain impiridones (ONC201 and ONC212) and the evidenced cross-talk between redox signalling and TRAIL activity we envisaged the synthesis of new ferrocene-containing hybrid compounds with impiridone core containing compounds carrying halogenated benzyl groups. The comparative in vitro assays of target compounds outlined are expected to disclose a new set of valuable structure–activity relationships that might be explored in the design of further members of impiridones with enhanced activity and cell selectivity.

### 2.1. Synthesis of the Reference and Hybrid Impiridones

**Scheme 1:**
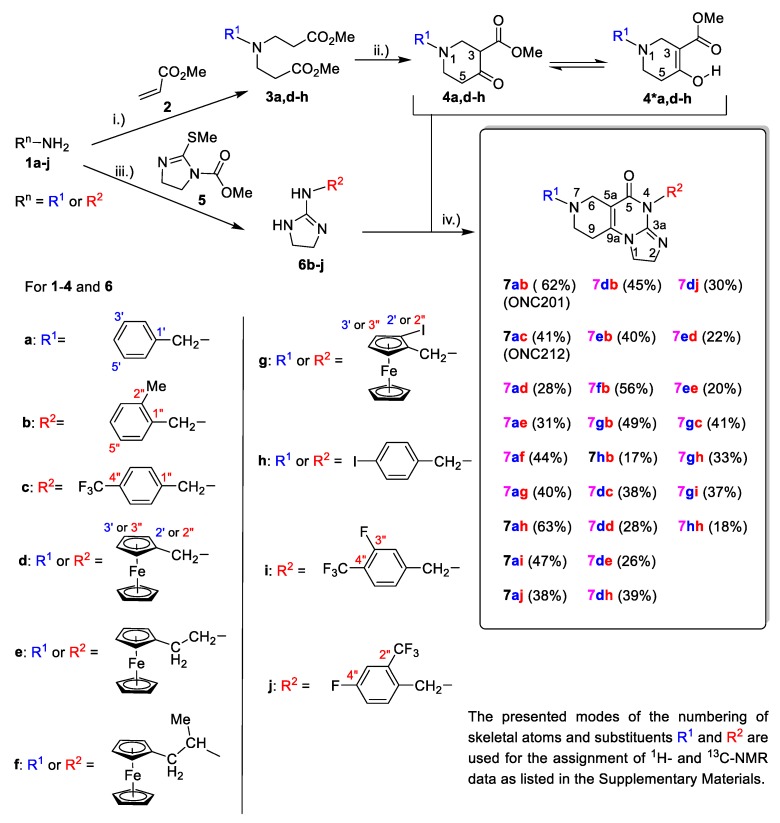
Synthetic route of novel ferrocene hybrids of ONC201 with compounds carrying halogenated benzyl groups. Reaction conditions: (i) MeOH, 25 °C, 24 h; (ii) NaH, THF, reflux, 2 h; (iii) AcOH, reflux, 20 h; (iv) NaOMe, MeOH, reflux, 12 h (the overall yields are presented).

Starting from the common primary amine precursors **1a**–**j** including ferrocene-based ones **1d**–**g** we elaborated a facile convergent synthetic route to novel impiridone hybrids type **7** allowing easy variation of substituents R^1^ and R^2^ (Scheme 1). On the one hand, the corresponding amine carrying R^1^ group was reacted with two equivalents of methylacrylate (**2**) in methanol at 25 °C affording diester **3** which, in crude form, was then cyclised by sodium hydride in THF at reflux temperature resulting in methoxycarbonyl-substituted piperidones tpye **4** that feature partial tautomerisation to the appropriate enol 4* as exemplified by the ^1^H-NMR and ^13^C-NMR spectral data of **4d** (see Supplementary Materials). The complementary amine component with the pending R^2^ group was coupled with the activated methyltioimidazoline **5** in boiling acetic acid to obtain cyclic guanidines type **6** [35].

In the final step, the targeted impiridones type **7** were obtained in moderate-to-good overall yields (Scheme 1) by the base-catalysed condensation of the selected pair of compounds **4** and **6** performed under standard conditions (NaOMe in MeOH at reflux temperature) [24]. Since the ferrocene-based amines **1d**–**g** are not commercially available, we accessed these reagents by well-established described synthetic routes [36,37,38,39].

### 2.2. NMR Analysis of the Skeletal Structure of Novel Impiridones

The skeletal structure and the substitution pattern of the novel impiridones were unambiguously confirmed by combined use of ^1^H- and ^13^C-NMR methods including 2D correlation measurements such as ^1^H-^13^C-HSQC, ^1^H-^13^C-HMBC, and ^1^H-^1^H-NOESY. The angular constitution of the tricyclic core is evidenced by the HMBC cross peak generated by the three-bond N4-CH_2_/C-5 correlation and the NOESY interaction involving the proximal methylene protons H-9 and H-1. In the ^1^H-NMR spectrum of **7ag**, due to the presence of adjacent planar chiral 2-iodoferrocenyl moiety in R^2^, the diastereotopic H_A_ and H_X_ protons of the N4-CH_2_ group give considerably separated doublets at 5.01 ppm and 4.76 ppm, respectively, while the minimal separation of the N7-CH_2_ signals (3.67 and 3.64 ppm) is in accord with the decreased desymmetrising effect of the more distant planar chiral fragment. It is of note that in the ^1^H-NMR spectra of **7gb**, **7gc**, **7gh**, and **7gi**, the planar chiral 2-iodoferrocenyl moiety in R^1^ causes highly significant AX-type split on the signal of H-6 protons (∆δ = 0.24 ppm for each), while no separation of N7-CH_2_ signal is discernible in spite of the close neighbourhood of the planar chiral element.

### 2.3. DFT Analysis of the Simplified Model Impiridones Carrying 2-Iodoferrocenylmethyl or Ferrocenylmethyl Group as R^1^-Substituent

**Figure 2:**
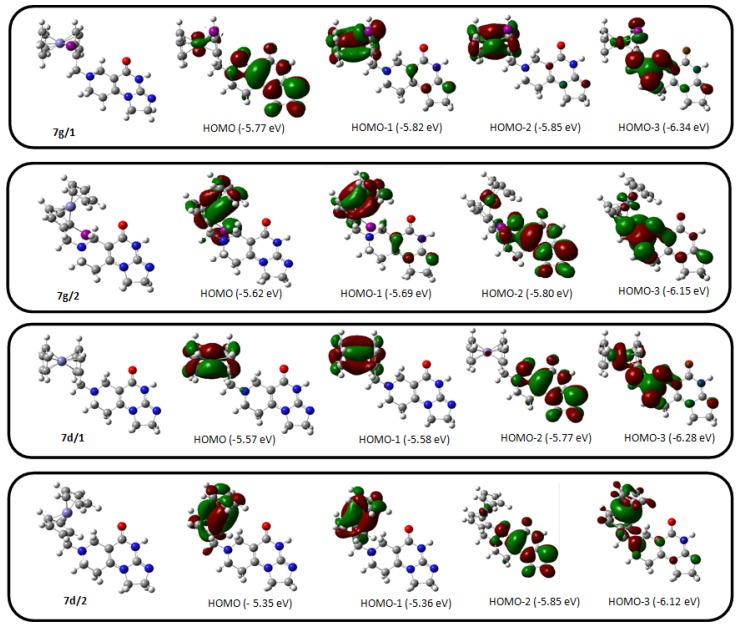
Optimised structures and four occupied MO’s of the stable rotamers of simplified N7-(2-iodoferrocenyl)/ferrocenylmethyl-substituted impiridone models **7g** and **7d**. HOMO: Highest Occupied Molecular Orbital.

Although at this initial stage of research, these compounds were synthesised and tested in racemic mixtures, from the aspect of their binding to a biological target, the characteristic pattern of the chemical shifts of the aforementioned diastereotopic proton pairs refer to the hindered rotation of the 2-iodoferrocenylmethyl group adopting a well-defined assembled position in the piperidine-region of the impiridone framework. In order to identify the conformation of the *N*-(2-iodoferrocenylmethyl)-piperidine segment with planar chirality, conformational chirality of the partly saturated heterocycle and the central chirality of N-4 atom, two rotamers of the simplified model **7g** (**7g**/**I** and **7g**/**II**: Figure 2) carrying equatorially positioned organometallic unit were subjected to comparative DFT analysis carried out by B3PW91 functional [40] using extended DGTZVP basis set [41]. The geometry optimisation of the conformers with axially positioned bulky 2-iodoferrocenylmethyl substituents were also attempted, but due to convergence problems no local minima could be identified on the potential energy surface. The equatorial orientation of the organometallic group can in principle be retained when the flip of the N-7 stereogenic centre is accompanied by simultaneous inversion of the tetrahydropyridine ring adopting enantiomeric half-chair conformations. The energetic data suggest that the population of rotamer **7g**/**1** seems to be highly dominant over that of **7g**/**2** (E(**7g**/**1**) − E(**7g**/**2**) = −3.84 kcal/mol) destabilised by the repulsion of the proximal iodine centre and the lone pair of N-7 atom. The dominant presence of this rotamer is supported by the characteristic NOE detected between H-5′ (on the 2-iodoferrocenyl moiety) and H-6_A_ separated by 3.693 Å as discernible on the optimised structure of **7g**/**1**. In order to get an insight into the effect of iodine in the organometallic moiety, the related optimised structures of two rotamers of the simplified ferrocenylmethyl model compound **7d** (**7d**/**1** and **7d**/**2**) were also identified by the B3PW91/DGTZVP method.

Their relative energetics (E(**7d**/**1**) − E(**7d**/**2**) = −1.44 kcal/mol) seems to allow more balanced population than that can be guessed for rotameric pairs of iodo analogues **7g**/**1** and **7g**/**2**. The relative propensity of the investigated compounds with ferrocene-based R^1^-substituents was indirectly assessed by the analysis of the filled frontier MO’s (HOMO, HOMO-1, HOMO-1, and HOMO-1) of the optimised rotamers of **7g**/**1**, **7g**/**2**, **7d**/**1**, and **7d**/**2** (Figure 2). First, it is worth to point out that contrary to ferrocene-centred HOMO’s of **7g**/**2**, **7d**/**1**, and **7d**/**2** the HOMO localised for the dominant iodine-containing rotamer **7g**/**1** with a minimal share on the ferrocene unit primarily seems to be associated with the basicity of the cyclic amidine part rather than with iron-promoted ROS generation. This characteristic HOMO-distribution certainly efficiently assists the formation of a strong H-bond with the corresponding functional groups in the binding site of a biological target. As our iodoferrocenylmethyl-substituted compounds have been prepared in racemic form, considering the increased importance of ultimate TRAIL-inducing binding to a biological target (certainly with chiral binding sites) relative to the role of ROS-generation, even at this point it might be anticipated that the cytotoxicity of a particular enantiomer must be significantly enhanced relative to those reflected by the IC_50_ values measured for **7gb**, **7gc**, **7gh**, and **7gi** as listed in Table 1. On the other hand, the ROS-induced oxidative stress, presumably operating in an orchestrated manner with the TRAIL mechanism as mentioned above, might play an important role in the effect of compounds containing unsubstituted ferrocenylalkyl-type R^1^ substituents. This view gains indirect support from the MO-analysis of rotamers **7d**/**1** and **7d**/**2** that disclosed practically exclusive share of the ferrocenyl group from the HOMO/HOMO-1 pairs with almost identical energy levels significantly higher than those calculated for **7g**/**l** and **7g**/**2** with iodoferrocenyl group (Figure 2). On the basis of these results, it also seems reasonable to assume a pronounced contribution of ROS-mediated oxidative stress to the measured cytotoxicity of the impiridones containing ferrocenyl units in both R^1^ and R^2^ substituents **7dd**, **7de**, **7ed**, and **7ee** (discussed below).

### 2.4. In Vitro Cytotoxicity Study of the Reference and Novel Impiridones

In order to obtain a preliminary insight into their mode of action all new compounds type **7** and references **7ab** (ONC201) and **7ac** (ONC212) were first evaluated for acute cytotoxic activity in vitro on human malignant cell lines HT-29 (human well-differentiated colon adenocarcinoma) and A2058 (human malignant melanoma with high invasiveness) by 3-(4,5-dimethylthiazol-2-yl)-2,5-diphenyltetrazolium bromide (MTT) tests with short incubation time (1 h) (Table 1/short-term treatment). As with the exception of 4-halobenzyl derivatives **7ah** and **7ai** as well as *bis*-ferrocenylalkyl-substituted impiridone **7ed**, the majority of the compounds produced only a limited and/or hardly reproducible cytotoxicity as reflected by the half maximal inhibitory concentration (IC_50_) and SD values. Therefore, we resorted to xCELLigence or alamar Blue methods employing prolonged treatment (72 h) of A2058 along with cell lines PANC-1 (human pancreatic carcinoma of ductal origin), COLO-205 (human colon adenocarcinoma), and EBC-1 (human lung squamous cell carcinoma) (Table 1/long-term treatment). The experimental details of the in vitro cytotoxicity tests are found in the Supplementary Materials.

The listed IC_50_ values (Table 1/long-term treatment) suggest that compounds bearing one ferrocenylalkyl group and benzyl- or 2-methylbenzyl substituent on either regions of the impiridone core (**7ad**, **7ae**, **7db**, and **7eb**) do not produce well-defined tumour growth inhibition featuring uncertain and much less effect on all investigated cell lines (particularly on PANC-1 and COLO-205) than their common organic reference **7ab** (ONC201). However, it must be pointed out that, probably at least partially due to ROS-mediated oxidative stress, *bis*-ferrocenylalkyl derivatives **7dd**, **7de**, **7ed**, and **7ee** display a much more pronounced long-term cytotoxic effect against A2058 cell line than do the organic impiridones with fluorinated R^2^ groups including ONC212 (**7ac**), the emblematic reference that produced improved preclinical efficacy on melanoma [26], as mentioned above in the Introduction. In this regard, it is of note that contrary to the majority of *mono*-ferrocenylalkyl-substituted impiridones (except for **7dc** carrying 4-trifluoromethylbenzyl group as R^2^-substituent rendering enhanced efficacy); *bis*-organometallics showed substantial activity against COLO-205 cell line. The data also indicate a clear general tendency that introduction of halo-substituted benzyl groups as R^2^ substituents leads to molecules having substantially enhanced cytotoxicity relative to their halogen-free counterparts. On the other hand, the replacement of N7-benzyl group (R^1^) for ferrocenylmethyl group gives rise to a significant increase in the cytotoxic activity against A-2058- and EBC-1 cell lines (refer to IC_50_ values: >25.0 μM (**7ac**) and 7.7 μM (**7dc**) on A-2058; 25.0 μM (**7ac**) and 8.0 μM (**7dc**) on EBC-1), while introduction of iodine in C-2′ position of the metallocene unit in R^1^-substituent induces a spectacular decrease in the efficacy on A-2058, COLO-205, and EBC-1 cell lines probably associated with a slight suppression of ROS-generation and/or binding discrimination of the planar chiral enantiomers (Table 1/long-term treatment). The IC_50_ data obtained after long-term treatment clearly indicate that the ferrocene-containing molecules display only low-to-moderate cytotoxicity on PANC-1, while except for polyfluorinated compound **7aj**, the halobenzyl derivatives proved to be highly active against this cell line in accord with the aforementioned precedencies described for ONC212 (**7ac**) [35]. Pointing to the importance of the substitution pattern in R^2^, **7ai**, with the same set of substituents far outperforms its isomeric counterpart **7aj** on all the investigated cell lines. It is of particular importance that the efficiency of this compound against PANC-1 and COLO-205 cell lines is similar to that produced by ONC212. Moreover, after short-term treatment, **7ai** proved to be more cytotoxic on HT-29 and A-2058 cell lines than the emblematic reference. Finally, it must be pointed out that this research identified **7ah**, the simple 4-iodobenzyl derivative as the most potent novel compound displaying similar or superior effects on the investigated cell lines to those produced by ONC212.

## 3. Materials and Methods

All chemicals (expect **1d**–**g**) were obtained from commercially available sources (Aldrich, St. Louis, MO, USA, Fluka) and used without further purification, expect solvents. Ferrocenylalkylamines **1d**–**g** were synthesised by well-established reported procedures: **1d** [37]; **1e** [38]; **1f** [39]; **1g** [40]. Methanol and triethylamine (TEA) were distilled from sodium, while THF was distilled from sodium benzophenone. Merck Kieselgel (SHIV Chemicals, Jalalpore, State Guajarat, India) (230–400 mesh, 60 Å) was used for flash column chromatography. The exact mass measurements were performed using a Thermo Scientific Q-Exactive Focus mass spectrometer (Daltonics GMBH, Bremen, Free Hanseatic City, Germany) in positive electrospray mode. The ^1^H and ^13^C-NMR spectra were recorded in DMSO-*d*_6_ or CDCl_3_ solution in 5 mm tubes at room temperature (RT), on a Bruker DRX-500 spectrometer (Bruker Biospin, Karlsruhe, Baden Württemberg, Germany) at 500 (^1^H) and 125 (^13^C) MHz, with the deuterium signal of the solvent as the lock and TMS as internal standard (^1^H, ^13^C). The 2D-COSY, NOESY, HSQC, and HMBC spectra were obtained by using the standard Bruker pulse programs (cosygpppqf (2D COSY with gradient pulses for selection and purge pulses before relaxation delay d1) for COSY, noesygpphpp (2D phase sensitive NOESY with gradient pulses in mixing time and purge pulses before relaxation delay d1 for NOESY), hsqcetgp (2D phase sensitive HSQC using Echo/Antiecho-TPPI gradient selection with decoupling during acquisition and using trim pulses in inept transfer) for HSQC and hmbcgpndqf (2D H-1/X HMBC optimized on long range couplings, no decoupling during acquisition using gradient pulses for selection) for HMBC, Bruker Biospin, Karlsruhe, Baden Württemberg, Germany). All calculations were carried out by using Gaussian 09 software package [42]. The optimised structures are available from the authors.

### 3.1. General Procedure for the Synthesis of Impiridones Type ***7***

#### 3.1.1. Synthesis of 2-(Methylthio)-4,5-Dihydro-1*H*-imidazole-1-carboxylate (**5**)

2-Methylthio-4,5-dihydroimidazolium iodide (12.21 g, 50 mmol) and triethylamine (TEA, 16 mL, 11.62 g, 115 mmol) were dissolved in DCM (50 mL). Methylchloroformate (5 mL, 6.12 g, 65 mmol) was added drop-wise to the solution previously cooled down to 0 °C. The reaction mixture was allowed to warm up to 25 °C and was stirred overnight. After addition of EtOAc (200 mL) and stirring for 15 min, the precipitated ammonium salts were filtered off and washed through with EtOAc (50 mL). The combined solution was evaporated to dryness. The solid residue was triturated with water filtered off and dried under vacuum to obtain **5** as white solid. Yield: 5.55 g (64%). The analytical and spectral data of a sample were practically identical to those reported by Jacob et al. [35].

#### 3.1.2. Synthesis of *N*-Aralkyl-4,5-dihydro-1*H*-imidazol-2-amine as Representative Example for the Preparation of the Cyclic Guanidines **6b**–**j**

To the solution of amine R^2^-NH_2_ (2 mmol) dissolved in a mixture of MeOH:AcOH (4 mL:1 mL) methyl 2-(methylthio)-4,5-dihydro-1*H*-imidazole-1-carboxylate (0.47g, 2.4 mmol) was added and the resulting solution was stirred at a reflux 20 h. After cooling down the reaction was concentrated in vacuo and the oily residue was dissolved in DCM (30 mL). The solution was washed with 3 M NaOH (10 mL), brine (10 mL), dried over Na_2_SO_4_, and evaporated to dryness. The colourless (or orange yellow) oil was crystallised from MeCN or ether and used without further purification for the cyclisation to impiridone framework.

#### 3.1.3. General Procedure for the Synthesis of Impiridones Type **7**

To the amine component R^1^-NH_2_ (3 mmol), dissolved in MeOH, methylacrylate (0.68 mL, 7.5 mmol) was added and the mixture was stirred for 24 h at room temperature, concentrated in vacuo and the obtained crude amine (**3a**,**d**–**h**) was dissolved in anhydrous THF (12 mL). Under Ar atmosphere NaH (0.36 g, 15 mmol) was added in small portions to the intensively stirred solution that previously was cooled down to 0 °C. The resulting suspension was stirred for additional 2 h at reflux temperature and concentrated to dryness under vacuum. The resulting solid residue containing the crude sodium salt of methoxycarbonylpiperidone (type **4**) was dissolved in anhydrous MeOH (15 mL). To this solution was added 2-(R^2^-NH)-substituted 3,4-dihydroimidazole of type **6** (3 mmol) prepared in separate steps as described above (Section 3.1.1 and 3.1.2). The basic solution was stirred at a reflux for 12 h, under Ar atmosphere then concentrated to dryness, and the solid residue was dissolved in dichloromethane (DCM, 60 mL). The solution was washed with brine (30 mL), dried over Na_2_SO_4_, filtered, and concentrated in vacuum. The solid/oily residue was subjected to subsequent flash column chromatography (over silica using DCM:MeOH 10:1 as eluent) and recrystallisation from methanol. The yields of the pure products type **7** presented on Scheme 1 were calculated from the initial amount (3 mmol) of the precursor amine R^1^-NH_2_.

Spectral characterisation (^1^H- and ^13^C-NMR data and HRMS) of the compounds type **7** can be found in the Supplementary Materials.

## 4. Conclusions

In order to extend the group of the analogues of the renowned TRAIL activators ONC201 and ONC212 and to establish novel, reasonably utilisable SAR to assess the contribution of oxidative stress to the in vitro cytotoxicity, we synthesised and tested a series of novel ferrocenylalkyl-substituted impiridones including benzyl- and halobenzyl-substituted compounds along with reasonably selected, while highly promising organic analogues as complementary models. Our synthetic strategy is based on the use of common, easily accessible ferrocenylalkylamine and benzylamine precursors that can be incorporated in both R^1^- and R^2^-substituents allowing for optional and selective functionalisation of the heterocyclic skeleton. Besides revealing cell-selectivity and a prolonged mechanism of action (long-term cytotoxicity) on the A-2058 cell line, the in vitro assays performed on five human malignant cell lines provided characteristic SAR that points to a possible role of ROS-mediated oxidative stress in the cytotoxic activity of the organometallic products. This view was supported by a preliminary DFT analysis of four filled frontier MO’s of selected simplified models. Finally it can be concluded that the optional chemoselective introduction of fine-tuned redox-active organometallic and halobenzyl groups into N4- and N7-positions seems to be a promising strategy for developing further impiridones of enhanced efficacy as a result of carefully designed implication of TRAIL-activation and ROS-mediated oxidative stress cooperating in an orchestrated manner to trigger signals finally leading to cell death. The presented research also identified a simple iodine-containing impiridone (R^1^ = benzyl; R^2^ = 4-iodobenzyl) as a highly potent antiproliferative agent with comparable or superior activity to that produced by ONC212 on the five investigated cell lines. As a continuation of our research aimed at collecting useful guidelines for the design of more efficient organometallic analogues, we envisage a study on the mechanism of action of the *bis*-ferrocenylalkyl-substituted impiridone **7de**, the compound found to be the most potent representative of the novel organometallics presented in this contribution.

## Figures and Tables

**Table 1:** In vitro cytotoxic effect of compounds **7ab**, **7ac**, and novel impiridones on various cell lines characterised by half maximal inhibitory concentration (IC_50_) ± Standard Deviation (SD) values.

			Short-Term Treatment ^a^		Long-Term Treatment ^b^			
	R^1^	R^2^	HT-29	A-2058	A-2058	PANC-1	COLO-205	EBC-1
**7ab**	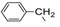	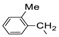	16.9 ± 8.6	27.6 ± 2.5	>25	1.7 ± 0.3	5.0 ± 2.9	7.0 ± 0.5
**7ac**	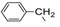	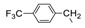	43.3 ± 38.6	35.1 ± 30.6	>25	0.16 ± 0.03	0.25 ± 0.03	25.0 ± 2.1
**7ad**	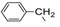	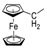	-	-	>10	>10	>10	>10
**7ae**	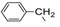	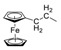	58.6 ± 18.6	-	>10	>25	>10	>10
**7af**	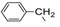	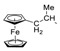	55.7 ± 3.8	45.4 ± 2.6	37.9 ± 15.3	36.6 ± 3.4	33.1 ± 6.8	28.9 ± 6.8
**7ag**	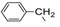	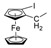	-	-	12.9 ± 1.6	>25	12.8 ± 1.7	~25 ± 5.9
**7ah**	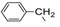	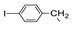	2.7 ± 1.1	8.8 ± 7.1	>25	0.3 ± 0.1	0.21 ± 0.05	1.0 ± 0.1
**7ai**	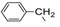	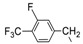	14.3 ± 7.8	14.2 ± 8.1	>25	0.28 ± 0.06	~0.25 ± 0.03	~25.0 ± 1.9
**7aj**	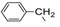	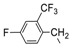	69.4 ± 7.8	>100	> 50	~50 ± 13.9	17.8 ± 1.0	>50
**7db**	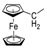	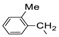	>100	>100	>10	>25	>10	>10
**7eb**	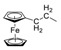	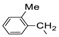	>100	>100	>25	>25	>25	>25
**7fb**	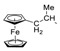	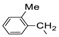	72.4 ± 13.0	52.0 ± 1.1	15.9 ± 1.8	38.0 ± 3.1	16.7 ± 3.0	24.3 ± 2.3
**7gb**	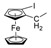	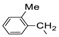	26.5 ± 15.2	36.3 ± 13.4	11.8 ± 0.7	>25	11.4 ± 0.1	15.7 ± 3.4
**7hb**	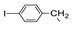	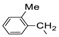	>100	12.8 ± 0.2	>50	21.6 ± 2.2	32.0 ± 1.4	18.0 ± 2.5
**7dc**	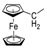	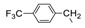	40.9 ± 2.4	28.1 ± 13.3	7.7 ± 1.2	21.4 ± 2.6	5.7 ± 0.9	8.0 ± 1.5
**7dd**	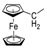	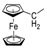	35.6 ± 0.5	31.2 ± 8.9	5.8 ± 0.7	>25.0	5.0 ± 3.1	>10
**7de**	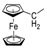	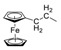	33.0 ± 23.3	20.9 ± 12.5	3.3 ± 0.3	16.3 ± 0.2	2.7 ± 0.1	11.1 ± 0.3
**7dh**	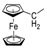	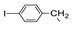	>100	57.2 ± 20.2	8.0 ± 0.7	36.8 ± 2.5	13.5 ± 1.0	15.7 ± 0.6
**7dj**	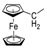	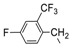	>100	42.0 ± 26.8	11.8 ± 0.4	>50	13.0 ± 3.2	17.1 ± 1.1
**7ed**	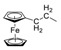	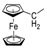	11.6 ± 3.5	13.5 ± 8.5	11.2 ± 5.6	>25	9.4 ± 1.6	13.1 ± 2.4
**7ee**	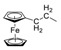	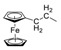	34.0 ± 9.9	17.6 ± 6.7	12.1 ± 4.9	>25	8.5 ± 0.9	>25
**7gc**	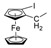	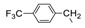	40.2 ± 5.3	28.7 ± 11.9	13.1 ± 0.9	22.2 ± 2.9	7.7 ± 2.2	12.8 ± 1.8
**7gh**	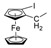	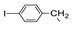	>100	49.8 ± 13.6	22.9 ± 2.5	>50	29.2 ± 2.3	28.2 ± 1.4
**7gi**	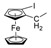	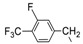	39.9 ± 9.8	32.3 ± 12.5	17.0 ± 1.3	19.9 ± 0.6	20.6 ± 0.6	18.3 ± 0.3
**7hh**	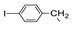	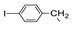	8.3 ± 0.8	-	31.6 ± 4.6	4.1 ± 0.2	2.3 ± 0.5	9.4 ± 3.8

^a^ Short-term (1 h) cytotoxic effects of the reference and hybrid impiridones were determined by 3-(4,5-dimethylthiazol-2-yl)-2,5-diphenyltetrazolium bromide (MTT)-assay after washing out the substances from the cells and a further 72 h of culturing. ^b^ The effect of long-term treatment (72 h) with the reference and hybrid impiridones was examined by xCELLigence System for PANC-1 cells or alamarBlue-assay for A-2058-, COLO-205- and EBC-1 cell lines.

## Supplementary Materials

The following are available online. ^1^H- and ^13^C-NMR data of a tautomeric mixture of **4d** and **4d***; ^1^H-and ^13^C-NMR data and HRMS of impiridones type **7**; the experimental details of the cell culturing and the in vitro assays.
