# Supplementary material for: Ferrocene-Containing Impiridone (ONC201) Hybrids: Synthesis, DFT Modelling, In Vitro Evaluation, and Structure–Activity Relationships
Source: Molecules. 2018 Sep 3;23(9):2248. doi: 10.3390/molecules23092248 (PMC6225426; doi:10.3390/molecules23092248)
Supplement: Supplementary file 1 [file molecules-23-02248-s001.pdf]

Supplementary Materials

Ferrocene-containing impiridone (ONC201) hybrids: synthesis, DFT modelling, *in vitro* evaluation and structure-activity relationships

Péter Bárány [1], Rita Oláh [2], Imre Kovács [1], Tamás Czuczi [1], Csenge Lilla Szabó [1], Angéla Takács [3], Eszter Lajkó [3], Orsolya Láng [3], László Kőhidai [3], Gitta Schlosser [1], Szilvia Bősze [2], Gábor Mező [2], Ferenc Hudecz [1,2] and Antal Csámpai [1,*]

1 Institute of Chemistry, Eötvös Loránd University (ELTE) Budapest Pázmány P. sétány 1/A, H-1117, Hungary; peterbarany@ceasar.elte.hu (P.B.); kimre950518@gmail.com (I.K); czuczi.tamas@gmail.com; (T.C.); szabo.csenge44@gmail.com; (C.L.Sz.); gitta.schlosser@gmail.com (G.S.); fhudecz@caesar.elte.hu (F.H.)

2 MTA-ELTE Research Group of Peptide Chemistry, Budapest Pázmány P. sétány 1/A, H-1117, Hungary; rita.olah.szabo@gmail.com (R.O.); szilvia.bosze@gmail.com (Sz.B.); gmezo@caesar.elte.hu (G.M.)

3 Department of Genetics, Cell- and Immunobiology, Semmelweis University, H-1089 Budapest, Nagyvárad tér 4, Hungary; angela.takacs1@gmail.com (A.T.); lajesz@gmail.com (E.L.); langorsi@gmail.com (L.O.); kohlasz2@gmail.com (L.K.)

* Correspondence: csampai@caesar.elte.hu; Tel.: +36-01-372-2500/6591

1. *^{1}H- and ^{13}C-NMR data of an 5:4 mixture of **4d** and its enol counterpart **4d*** detected in DMSO-d_6 at 25 ^{o}C.*

Methyl 1-ferrocenylmethyl-4-oxopiperidine-3-carboxylate 4d: ^{1}H-NMR: 4.10 [s, η^5-$C_5\underline{H}_5$ (Fc)]; 4.09 [br ~s, H-3′,4′ (Fc)]; 4.08 and 4.06 [br ~s′s, H-2′ and H5′ (Fc)]; 3.56 (s, $CO_2C\underline{H}_3$); 3.51 (dd, *J*=9.0 Hz and 5.2 Hz, H-3); 3.47 and 3.44 (A and B part of an AB spin system, J_{AB}=13.3 Hz, N-$C\underline{H}_AH_X$ and N-$CH_A\underline{H}_X$); 2.90 (dd, *J*=11.7 Hz and 5.2 Hz, H-2$_{equatorial}$); 2.77 (br ~t, J~10 Hz, H-6$_{axial}$); 2.68 (dd, *J*=11.7 Hz and 9.0 Hz, H-2$_{axial}$); 2.46 (overlapped by the DMSO-d_5 signal of the solvent, detected by 2D-HSQC measurement, H-6$_{equatorial}$); 2.26-2.22 (m, H-5$_{equatorial}$ and H-5$_{axial}$). ^{13}C-NMR: 204.7 (C-4) 169.6 ($\underline{C}O_2CH_3$); 82.9 [C-1′ (Fc)]; 70.25 [C-3′,4′ (Fc)]; 68.9 [coalesced with the same signal of the enol **4d***, η^5-$\underline{C}_5H_5$ (Fc)]; 70.25 [C-3′,4′ (Fc)]; 68.25 and 68.21 [C-2′ and C-3′ (Fc)]; 56.2 (N-$\underline{C}H_AH_X$); 56.0 (C-3); 54.4 (C-2); 52.3 (C-6); 52.2 ($CO_2\underline{C}H_3$); 29.4 (C-5).

Methyl 1-ferrocenylmethyl-4-hydroxy-1,2,5,6-tetrahydropyridine-3-carboxylate 4d*: ^{1}H-NMR: 11.68 (s, O$\underline{H}$, enol); 4.13 [br ~t, *J*~2 Hz, H-2′,5′ (Fc)]; 4.08 [s, η^5-$C_5\underline{H}_5$ (Fc)]; 4.07 [br ~t, *J*~2 Hz, H-3′,4′ (Fc)]; 3.63 (s, $CO_2C\underline{H}_3$); 3.39 (N-$C\underline{H}_2$); 2.92 (br s, H-2); 2.52 (t, *J*=6.8 Hz, H-6); 2.33 (t, *J*=6.8 Hz, H-6). ^{13}C-NMR: 171.2 (C-4); 170.5 ($\underline{C}O_2CH_3$); 96.7 (C-3); 83.4 [C-1′ (Fc)]; 70.21 [C-3′,4′ (Fc)]; 68.9 [coalesced with the same signal of the oxo tautomer **4d**, η^5-$\underline{C}_5H_5$ (Fc)]; 68.16 [C-2′,5′ (Fc)]; 57.0 (N-$\underline{C}H_2$); 52.0 ($CO_2\underline{C}H_3$); 48.8 (C-2); 48.5 (C-6); 32.6 (C-5).

2. *Characterisation (^{1}H- and ^{13}C NMR data and HRMS) of the compounds type **7***

- **7-Benzyl-4-(2-methylbenzyl)-2,4,6,7,8,9-hexahydroimidazo[1,2-a]pyrido[3,4-e]pyrimidin-5(1*H*)-one (7ab)**: ^{1}H-NMR ($CDCl_3$): 7.32-7.29 [m, 4H, H-2′,3′5′,6′(R^1)]; 7.23 [m, H-4′,1H (R^1)], 7.12-7.08 [m, 4H, H-2″-6″ (R^2)]; 5.02 (s, 2H, N4-$C\underline{H}_2$); 3.89 (A part of an A_2B_2 spin system, *J*=9.8 Hz, 2H, H-2); 3.84 (B part of an A_2B_2 spin system, *J*=9.8 Hz, 2H, H-1); 3.64 (s, 2H, N7-$C\underline{H}_2$); 3.29 (br s, 2H, H-6); 2.65 (t, *J*=5.7 Hz, 2H, H-8); 2.46 (t, *J*=5.7 Hz, 2H, H-9); 2.37 [s, 3H, $C\underline{H}_3$ (R^2)]. ^{13}C-NMR ($CDCl_3$): 161.6 (C-5); 153.4 (C-3a); 145.7 (C-9a); 137.8 [C-1′ (R^1)]; 135.5 [C-1″ (R^2)]; 134.3 [C-2″ (R^2)]; 130.2 [C-3″ (R^2)]; 129.1 [C-2′,6′ (R^1)]; 128.4 [C-3′,5′ (R^1)]; 127.4 [C-4′ (R^1)]; 129.1 [C-2′,6′ (R^1)]; 126.8 [C-5″ (R^2)]; 125.9 [C-4″ (R^2)];

125.3 [C-6″ (R^2)]; 102.1 (C-5a); 62.3 (N7-$\underline{C}H_2$); 50.7 (C-2); 49.7 (C-6); 48.3 (C-8); 46.8 (C-1); 43.2 (N4-$\underline{C}H_2$); 27.0 (C-9); 19.3 [$\underline{C}H_3$ (R^2)]. HRMS exact mass calculated for $C_{25}H_{27}N_4O$: 386.2107; found for $[MH]^+$: 387.2171.

- **7-Benzyl-4-(4-trifluoromethylbenzyl)-2,4,6,7,8,9-hexahydroimidazo[1,2-a]pyrido[3,4-e]pyrimidin-5(1*H*)-one (7ac)**: ^{1}H-NMR ($CDCl_3$): 7.51 and 7.50 [A and B part of an AA′BB′ spin system, J_{AB}=8.9 Hz, 2x2H, H-3″,5″ and H-2″,6″, resp. (R^2)]; 7.31-7.26 [m, 4H, H-2′,3′5′,6′(R^1)]; 7.23 [~tt, *J*~7 Hz and ~2 Hz, 1H, H-4′ (R^1)]; 5.06 (s, 2H, N4-C$\underline{H_2}$); 3.86 (s, 4H, H-1 and H-2); 3.63 (N7-C$\underline{H_2}$); 3.26 (br s, 2H, H-6]; 2.63 (t, 2H, *J*=5.7 Hz, H-8); 2.43 (t, 2H, *J*=5.7 Hz, H-9). ^{13}C-NMR ($CDCl_3$): 161.5 (C-5); 152.9 (C-3a); 146.0 (C-9a); 140.9 [C1″ (R^2)]; 137.8 [C-1′ (R^1)]; 129.6 [qa, *J*=32.2 Hz, C-4″ (R^2)]; 129.1 [C-2′,6′ (R^1)]; 128.7 [C-2″,6″ (R^2)]; 128.4 [C-3′,5′ (R^1)]; 127.4 [C-4′ (R^1)]; 125.3 [qa, *J*=3.9 Hz, C-3″,5″ (R^2)]; 124.7 [qa, *J*=272.5 Hz, C-3″,5″ (R^2)]; 101.9 (C-5a); 62.3 (N7-$\underline{C}H_2$); 50.6 (C-2); 50.0 (C-6); 48.2 (C-8); 47.0 (C-1); 45.0 (N4-$\underline{C}H_2$); 26.9 (C-9). HRMS exact mass calculated for $C_{25}H_{24}F_3N_4O$: 440.1824; found for $[MH]^+$: 441.1886.

- **7-Benzyl-4-(3-fluoro-4-trifluoromethylbenzyl)-2,4,6,7,8,9-hexahydroimidazo[1,2-a]pyrido[3,4-e]pyrimidin-5(1*H*)-one (7ai)**: ^{1}H-NMR ($CDCl_3$): 7.47 [t, *J*=7.6 Hz, 1H, H-5″ (R^2)]; 7.31-7.22 [overlapping m′, 7H, H-2′-6′(R^1) and H-2″,6″(R^2)]; 5.03 (s, 2H, N4-C$\underline{H_2}$); 3.87 (s, 4H, H-1 and H-2); 3.64 (N7-C$\underline{H_2}$); 3.26 (br s, 2H, H-6]; 2.64 (t, 2H, *J*=5.7 Hz, H-8); 2.46 (t, 2H, *J*=5.7 Hz, H-9). ^{13}C-NMR ($CDCl_3$): 161.3 (C-5); 159.8 [dqa, *J*=272.5 Hz and 1.4 Hz, C-3″ (R^2)]; 152.9 (C-3a); 146.2 (C-9a); 143.8 [d, *J*=7.0 Hz, C1″ (R^2)]; 137.6 [C-1′ (R^1)]; 129.1 [C-2′,6′ (R^1)]; 128.4 [C-3′,5′ (R^1)]; 127.4 [C-4′ (R^1)]; 127.0 [qad, *J*=4.3 Hz and 1.5 Hz, C-5″ (R^2)]; 124.0 [d, *J*=3.3 Hz, C-5″ (R^2)]; 122.7 [qad, *J*=272.5 Hz and 1.4 Hz, $\underline{C}F_3$ (R^2)]; 117.2 [qad, *J*=33.0 Hz and 12.4 Hz, C-4″ (R^2)]; 116.6 [d, *J*=21.0 Hz, C-2″ (R^2)]; 101.9 (C-5a); 62.3 (N7-$\underline{C}H_2$); 50.6 (C-2); 49.5 (C-6); 48.2 (C-8); 47.0 (C-1); 44.6 (N4-$\underline{C}H_2$); 26.9 (C-9). HRMS exact mass calculated for $C_{25}H_{23}F_4N_4O$: 458.1730; found for $[MH]^+$: 459.1793.

- **4-Benzyl-7-ferrocenylmethyl-2,4,6,7,8,9-hexahydroimidazo[1,2-a]pyrido[3,4-e]pyrimidin-5(1*H*)-one (7ad)**: ^{1}H-NMR (DMSO-d_6): 7.30-7.24 [m, 4H, H-2′,3′5′,6′(R^1)]; 7.21 [tt, *J*=7.2 Hz and 2.1 Hz, 1H, H-4′ (R^1)]; 4.60 (s, 2H, N4-C$\underline{H_2}$); 4.23 [t, *J*=2.0 Hz, 2H, H-2″,5″ (R^2)]; 4.12 [s, 5H, η^5-$C_5\underline{H_5}$ (R^2)]; 3.99 [t, *J*=2.0 Hz, 2H, H-3″,4″ (R^2)]; 3.84 (t, *J*=9.4 Hz, 2H, H-2); 3.68 (t, *J*=9.4 Hz, 2H, H-1); 3.54 (s, 2H, N7-C$\underline{H_2}$); 2.93 (br s, 2H, H-6]; 2.54 (t, 2H, *J*=5.7 Hz, H-8); 2.41 (t, 2H, *J*=5.7 Hz, H-9). ^{13}C-NMR (DMSO-d_6): 160.8 (C-5); 152.2 (C-3a); 147.2 (C-9a); 138.7 [C-1′ (R^1)]; 129.2 [C-2′,6′ (R^1)]; 128.7 [C-3′,5′ (R^1)]; 127.5 [C-4′ (R^1)]; 99.8 (C-5a); 83.2 [C-1″ (R^2)]; 70.4 [C-2″,5″ (R^2)]; 68.8 [η^5-$\underline{C_5}H_5$ (R^2)]; 67.9 [C-3″,4″ (R^2)]; 61.7 (N7-$\underline{C}H_2$); 50.6 (C-2); 49.2 (C-6); 48.8 (C-8); 46.6 (C-1); 40.7 (N4-$\underline{C}H_2$); 26.2 (C-9). HRMS exact mass calculated for $C_{27}H_{28}FeN_4O$: 480.1613; found for $[MH]^+$: 481.1672.

- **4-Benzyl-7-(2-ferrocenylethyl)-2,4,6,7,8,9-hexahydroimidazo[1,2-a]pyrido[3,4-e]pyrimidin-5(1*H*)-one (7ae)**: ^{1}H-NMR (DMSO-d_6): 7.30-7.24 [m, 4H, H-2′,3′5′,6′(R^1)]; 7.21 [tt, *J*=7.2 Hz and 2.1 Hz, 1H, H-4′ (R^1)]; 4.14 [s, 5H, η^5-$C_5\underline{H_5}$ (R^2)]; 4.05 [t, *J*=2.0 Hz, 2H, H-2″,5″ (R^2)]; 4.01 [t, *J*=2.0 Hz, 2H, H-3″,4″ (R^2)]; 3.91 (m, 2H, N4-C$\underline{H_2}$); 3.88 (t, *J*=9.5 Hz, 2H, H-2); 3.70 (t, *J*=9.5 Hz, 2H, H-1); 2.98 (br s, 2H, H-6]; 2.59 (t, 2H, *J*=5.7 Hz, H-8); 2.47 (t, 2H, *J*=5.7 Hz, H-9); 2.44 [m, 2H, C-C$\underline{H_2}$ (R^2)]. ^{13}C-NMR (DMSO-d_6): 161.1 (C-5); 152.3 (C-3a); 147.2 (C-9a); 138.7 [C-1′ (R^1)]; 129.2 [C-2′,6′ (R^1)]; 128.7 [C-3′,5′ (R^1)]; 127.5 [C-4′ (R^1)]; 100.0 (C-5a); 85.7 [C-1″ (R^2)]; 68.8 [η^5-$\underline{C_5}H_5$ (R^2)]; 67.8 [C-2″,5″ (R^2)]; 67.4 [C-3″,4″ (R^2)]; 61.8 (N7-$\underline{C}H_2$); 50.6 (C-2); 49.2 (C-6); 48.9 (C-8); 46.6 (C-1); 42.0 (N4-$\underline{C}H_2$); 26.2 [two coalesced lines C-9 and C-$\underline{C}H_2$ (R^2)]. HRMS exact mass calculated for $C_{28}H_{30}FeN_4O$: 494.1769; found for $[MH]^+$: 495,1829.

- **(±)-7-Benzyl-4-(1-ferrocenylpropan-2-yl)-2,4,6,7,8,9-hexahydroimidazo[1,2-a]pyrido[3,4-e]pyrimidin-5(1*H*)-one (7af)**: ^{1}H-NMR ($CDCl_3$): 7.30-7.27 [overlapping m′s, 4H, H-2′,3′,5′,6′ (R^1)]; 7.24 [m, 1H, H-4′ (R^1)]; ~4.8 (very br s, 1H, N4-C$\underline{H}$); 4.04 [s, 5H, η^5-$C_5\underline{H_5}$ (R^2)]; 4.02 [br s, 2H, H-2″,5″ (R^2)]; 3.97 [br s, 2H, H-3″,4″ (R^2)]; 3.63 (s, 2H, N7-C$\underline{H_2}$); 3.83-3.75 (overlapping m′s, 4H, H-1 and H-2); 3.24 (br s, 2H, H-6); 3.05 (dd, *J*=14.0 Hz and 7.2 Hz, 1H C-C$\underline{H_A}H_X$ (R^2)]; 2.90 (dd, *J*=14.0 Hz and 7.7 Hz, 1H C-C$H_A\underline{H_X}$ (R^2)]; 2.61 (t, *J*=5.7 Hz, 2H, H-8); 2.38 (t, *J*=5.7 Hz, 2H, H-9); 1.36 (d, J=6.8, 3H, C$\underline{H_3}$ (R^2)]. ^{13}C-NMR ($CDCl_3$): 162.1 (C-5); 152.8 (C-3a); 145.0 (C-9a); 137.8 [C-1′ (R^1)]; 129.1 [C-2′,6′ (R^1)]; 128.4 [C-3′,5′ (R^1)]; 127.3 [C-4′ (R^1)]; 102.5 (C-5a); 85.7 (C-1″ (R^2)]; 69.2 and 68.7 [C-2″ and C-5″ (R^2)]; 68.5 [η^5-$\underline{C_5}H_5$ (R^2)]; 67.4 and 67.3 [C-3″ and C-4″ (R^2)]; 62.3 (N7-$\underline{C}H_2$); ~55 (br s, N4-$\underline{C}H_2$); 50.4 (C-2); 49.5 (C-6); 48.2

(C-8); 46.5 (C-1); 33.2 [C-C̲H$_A$H$_X$ (R[2])]; 26.7 (C-9); 16.7 [C̲H$_3$ (R[2])]. HRMS exact mass calculated for $C_{29}H_{32}FeN_4O$: 508.1926; found for $[MH]^+$: 509.1990.

- **7-Benzyl-4-(4-iodophenylmethyl)-2,4,6,7,8,9-hexahydroimidazo[1,2-a]pyrido[3,4-e]pyrimidin-5(1*H*)-one** (**7ah**): ^{1}H-NMR ($CDCl_3$): 7.56 (d, *J*=8.4 Hz, 2H, H-3″,5″ (R[2])]; 7.31-7.26 [overlapping m's, 4H, H-2′,3′,5′,6′ (R[1])]; 7.23 [tt, *J*=7.2 Hz and 2.5 Hz, 1H, H-4′ (R[1])]; 7.17 (d, *J*=8.4 Hz, 2H, H-2″,6″ (R[2])]; 4.93 (s, 2H, N4-CH̲$_2$); 3.85 (A part of an A_2B_2 spin system, *J*=9.6 Hz, 2H, H-2); 3.83 (B part of an A_2B_2 spin system, *J*=9.6 Hz, 2H, H-1); 3.62 (s, N7-CH̲$_2$); 3.25 (br s, 2H, H-6); 2.61 (t, *J*=5.7 Hz, 2H, H-8); 2.41 (t, *J*=5.7 Hz, 2H, H-9). ^{13}C-NMR ($CDCl_3$): 161.4 (C-5); 152.9 (C-3a); 145.8 (C-9a); 137.6 [C-1′ (R[1])]; 137.3 [C-3″,5″ (R[2])]; 136.6 [C-1″ (R[2])]; 130.7 [C-2″,6″ (R[2])]; 129.1 [C-2′,6′ (R[1])]; 128.4 [C-3′,5′ (R[1])]; 127.4 [C-4′ (R[1])]; 102.0 (C-5a); 93.0 [C-4″ (R[2])]; 62.3 (N7-C̲H$_2$); 50.5 (C-2); 49.5 (C-6); 48.2 (C-8); 46.9 (C-1); 44.9 (N4-C̲H$_2$); 26.8 (C-9). HRMS exact mass calculated for $C_{23}H_{23}IN_4O$: 498.0917; found for $[MH]^+$: 499.0980.

- **4,7-Bis-(ferrocenylmethyl)-2,4,6,7,8,9-hexahydroimidazo[1,2-a]pyrido[3,4-e]pyrimidin-5(1*H*)-one** (**7dd**): ^{1}H-NMR ($CDCl_3$): 4.76 (s, 2H, N4-CH̲$_2$); 4.40 [~t, *J*~2 Hz, 2H, H-2″,5″ (R[2])]; 4,15 [br ~s, 2H, H-2′,5′ (R[1])]; 4.12 [s, 5H, η^5-C$_5$H̲$_5$ (R[2])]; 4.08 [two overlapping s's, 7H, H-3′,4′ and η^5-C$_5$H̲$_5$ (R[1])]; 4.02 [~t, *J*~2 Hz, 2H, H-3″,4″ (R[2])]; 3.87 (t, *J*=9.5 Hz, 2H, H-1); 3.75 (t, *J*=9.5 Hz, 2H, H-2); 3.48 (s, 2H, N7-CH̲$_2$); 3.17 (br s, 2H, H-6); 2.54 (t, *J*=5.7 Hz, 2H, H-8); 2.32 (t, *J*=5.7 Hz, 2H, H-9). ^{13}C-NMR ($CDCl_3$): 160.9 (C-5); 152.8 (C-3a); 145.1 (C-9a); 102.5 (C-5a); 82.5 [C-1′, (R[1])]; 82.3 [C-1″, (R[2])]; 70.5 [C-2″,5″, (R[2])]; 70.2 [C-2′,5′, (R[1])]; 68.5 [two coalesced lines, η^5-C̲$_5$H$_5$ (R[1] and R[2])]; 68.2 [C-3′,4′ (R[1])]; 67.9 [C-3″,4″ (R[2])]; 57.5 (N7-C̲H$_2$); 50.6 (C-2); 48.7 (C-6); 47.8 (C-8); 46.8 (C-1); 41.2 (N4-C̲H$_2$); 26.5 (C-9). HRMS exact mass calculated for $C_{31}H_{32}Fe_2N_4O$: 588.1275; found for $[MH]^+$: 509.1990.

- **7-Ferrocenylmethyl-4-(4-iodobenzyl)-2,4,6,7,8,9-hexahydroimidazo[1,2-a]pyrido[3,4-e]pyrimidin-5(1*H*)-one** (**7dh**): ^{1}H-NMR ($CDCl_3$): 7.55 [d, *J*=8.1 Hz, 2H, H-3″,5″ (R[2])]; 7.15 [d, *J*=8.1 Hz, 2H, H-2″,6″ (R[2])]; 4.16 [t, *J*=2.0 Hz, 2H, H-2′,5′ (R[1])]; 4.07 [s, 7H, H-3′,4′ and η^5-C$_5$H̲$_5$ (R[1])]; 4.93 (s, 2H, N4-CH̲$_2$); 3.82 (A part of an A_2B_2 spin system, *J*=9.8 Hz, 2H, H-2); 3.79 (B part of an A_2B_2 spin system, *J*=9.8 Hz, 2H, H-1); 3.49 (s, 2H, N7-CH̲$_2$); 3.19 (br s, 2H, H-6); 2.58 (t, *J*=5.7 Hz, 2H, H-8); 2.38 (t, *J*=5.7 Hz, 2H, H-9). ^{13}C-NMR ($CDCl_3$): 161.5 (C-5); 152.9 (C-3a); 145.6 (C-9a); 137.3 [C-3″,5″ (R[2])]; 136.6 [C-1″ (R[2])]; 130.6 [C-2″,6″ (R[2])]; 101.9 (C-5a); 93.0 [C-4″ (R[2])]; 82.2 [C-1′ (R[1])]; 70.2 [C-2′,5′ (R[1])]; 68.6 [η^5-C̲$_5$H$_5$ (R[1])]; 68.3 [C-3′,4′ (R[1])]; 57.5 (N7-C̲H$_2$); 50.6 (C-2); 48.7 (C-6); 47.8 (C-8); 46.8 (C-1); 44.8 (N4-C̲H$_2$); 26.7 (C-9). HRMS exact mass calculated for $C_{27}H_{27}FeIN_4O$: 606.0579; found for $[MH]^+$: 607,0630.

- **7-Ferrocenylmethyl-4-(2-trifluoromethyl-4-fluorobenzyl)-2,4,6,7,8,9-hexahydroimidazo[1,2-a]pyrido[3,4-e]pyrimidin-5(1*H*)-one** (**7dj**): ^{1}H-NMR ($CDCl_3$): 7.31 [dd, *J*=8.9 Hz and 2.4 Hz, 1H, H-3″(R[2])]; 7.08 [td, *J*=8.2 Hz and 2.4 Hz, 1H, H-5″(R[2])]; 7.02 [dd, *J*=8.2 Hz and 5.2 Hz, 1H, H-6″(R[2])]; 5.20 (s, 2H, N4-CH̲$_2$); 4.16 [t, *J*=1.9 Hz, 2H, H-2′,5′(R[1])]; 4.12 [s, 5H, η^5-C$_5$H̲$_5$ (R[1])]; 4.09 [t, *J*=1.9 Hz, 2H, H-3′,4′(R[1])]; 3.86 (A part of an A_2B_2 spin system, *J*=9.7 Hz, 2H, H-2); 3.82 (B part of an A_2B_2 spin system, *J*=9.7 Hz, 2H, H-1); 3.51 (s, 2H, N7-CH̲$_2$); 3.21 (br s, 2H, H-6); 2.63 (t, *J*=5.7 Hz, 2H, H-8); 2.46 (t, *J*=5.7 Hz, 2H, H-9). ^{13}C-NMR ($CDCl_3$): 161.2 (C-5); 161.0 [d, *J*=247.2 Hz, C-4″ (R[2])]; 153.2 (C-3a); 146.3 (C-9a); 130.7 [br ~s, C-1″ (R[2])]; 129.2 [dqa, *J*=32.0 Hz and 7.8 Hz, C-2″ (R[2])]; 127.9 [d, *J*=7.9 Hz, C-6″ (R[2])]; 123.5 [qa, *J*=276.0 Hz, C̲F$_3$ (R[2])]; 118.7 [d, *J*=21.0 Hz, C-5″ (R[2])]; 113.9 [dqa, *J*=25.2 Hz and 5.9 Hz, C-3″ (R[2])]; 101.9 (C-5a); 82.2 [C-1′ (R[1])]; 70.2 [C-2′,5′ (R[1])]; 68.6 [η^5-C̲$_5$H$_5$ (R[1])]; 68.4 [C-3′,4′ (R[1])]; 57.8 (N7-C̲H$_2$); 50.6 (C-2); 48.7 (C-6); 47.9 (C-8); 46.8 (C-1); 42.3 (qa, *J*=3.4 Hz, N4-C̲H$_2$); 26.7 (C-9). HRMS exact mass calculated for $C_{28}H_{26}F_4FeN_4O$: 566.1392; found for $[MH]^+$: 567.1451.

- **7-Benzyl-4-(2-trifluoromethyl-4-fluorobenzyl)-2,4,6,7,8,9-hexahydroimidazo[1,2-a]pyrido[3,4-e]pyrimidin-5(1*H*)-one** (**7aj**): ^{1}H-NMR ($CDCl_3$): 7.32 [dd, *J*=9.0 Hz and 2.3 Hz, 1H, H-3″(R[2])]; 7.30-7.28 [m, 4H, H-2′,3′,5′,6′ (R[1])]; 7.24 [m, 1H, H-4′ (R[1])]; 7.10 [td, *J*=8.3 Hz and 2.3 Hz, 1H, H-5″(R[2])]; 7.07 [dd, *J*=8.3 Hz and 5.2 Hz, 1H, H-6″(R[2])]; 5.22 (s, 2H, N4-CH̲$_2$); 3.91 (A part of an A_2B_2 spin system, *J*=9.5 Hz, 2H, H-2); 3.86 (B part of an A_2B_2 spin system, *J*=9.5 Hz, 2H, H-1); 3.64 (s, 2H, N7-CH̲$_2$); 3.27 (br s, 2H, H-6); 2.68 (t, *J*=5.8 Hz, 2H, H-8); 2.50 (t, *J*=5.8 Hz, 2H, H-9). ^{13}C-NMR ($CDCl_3$): 161.2 (C-5); 161.0 [d, *J*=247.4 Hz, C-4″ (R[2])]; 153.1 (C-3a); 146.3 (C-9a);137.5 [C-1′ (R[1])]; 130.7 [br ~s, C-1″ (R[2])];

129.3 [dqa, *J*=32.0 Hz and 7.8 Hz, C-2″ (R^2)]; 129.2 [C-2′,6′ (R^1)]; 128.5 [C-3′,5′ (R^1)]; 127.9 [d, *J*=7.9 Hz, C-6″ (R^2)]; 127.4 [C-4′ (R^1)]; 123.5 [qa, *J*=275.5 Hz, CF_3 (R^2)]; 118.7 [d, *J*=20.9 Hz, C-5″ (R^2)]; 113.9 [dqa, *J*=25.2 Hz and 5.9 Hz, C-3″ (R^2)]; 101.9 (C-5a); 62.4 (N7-CH_2); 50.6 (C-2); 49.4 (C-6); 48.3 (C-8); 47.1 (C-1); 42.2 (qa, *J*=3.4 Hz, N4-CH_2); 26.9 (C-9). HRMS exact mass calculated for $C_{25}H_{23}F_4N_4O$: 458.1730; found for $[MH]^+$: 459.1790.

- **7-(2-Ferrocenylethyl)-4-(2-methylbenzyl)-2,4,6,7,8,9-hexahydroimidazo[1,2-a]pyrido[3,4-e]pyrimidin-5(1*H*)-one** (**7eb**): ^{1}H-NMR ($CDCl_3$): 7.12-7.05 [m, 3H, H-3″-5″ (R^2)]; 7.03 [m, 1H, H-6″ (R^2)]; 5.04 (s, N4-CH_2); 4.07 [s, 5H, η^5-C_5H_5 (R^1)]; 4.05 [br s, 2H, H-2′,5′ (R^1)]; 4.03 [br s, 2H, H-3′,4′ (R^1)]; 3.88 (A part of an A_2B_2 spin system, *J*=9.5 Hz, 2H, H-2); 3.86 (B part of an A_2B_2 spin system, *J*=9.5 Hz, 2H, H-1); 3.31 (br s, 2H, H-6); 2.71 (t, *J*=5.8 Hz, 2H, H-8); 2.65 (m, 2H, N7-CH_2); 2.57 [m, 2H, C-CH_2 (R^1)]; 2.52 (t, *J*=5.8 Hz, 2H, H-9); 2.37 [s, 3H, CH_3 (R^2)]. ^{13}C-NMR ($CDCl_3$): 161.2 (C-5); 153.3 (C-3a); 145.6 (C-9a); 135.6 [C-1″ (R^2)]; 134.3 [C-2″ (R^2)]; 130.2 [C-3″ (R^2)]; 126.8 [C-5″ (R^2)]; 125.9 [C-4″ (R^2)]; 125.3 [C-6″ (R^2)]; 101.9 (C-5a); 86.3 [C-1′ (R^1)]; 68.6 [η^5-C_5H_5 (R^1)]; 68.2 [C-2′,5′ (R^1)]; 67.3 [C-2′,5′ (R^1)]; 59.4 (N7-CH_2); 50.6 (C-2); 49.4 (C-6); 49.1 (C-8); 47.0 (C-1); 43.2 (N4-CH_2); 26.9 (C-9); 19.3 [CH_3 (R^2)]. HRMS exact mass calculated for $C_{29}H_{32}FeN_4O$: 508.1926; found for $[MH]^+$: 509.1985.

- **4,7-Bis-(2-ferrocenylethyl)-2,4,6,7,8,9-hexahydroimidazo[1,2-a]pyrido[3,4-e]pyrimidin-5(1*H*)-one** (**7ee**): ^{1}H-NMR ($CDCl_3$): 4.76 (s, 2H, N4-CH_2); 4.13 [s, 5H, η^5-C_5H_5 (R^2)]; 4.11 [t, *J*=2.2 Hz, 2H, H-2″,5″ (R^2)]; 4.08 [s, 5H, η^5-C_5H_5 (R^1)]; 4.06 [t, *J*=2.2 Hz, 2H, H-2′,5 (R^1)]; 4.06 [t, *J*=2.2 Hz, 2H, H-2′,5′ (R^1)]; 4.04-4.01 [overlapping m′s, 6H, H-3′4′ (R^1), H-3″,4″ (R^2) and N4-CH_2]; 4.01 [t, *J*=2.2 Hz, 2H, H-2″,5″, (R^2)]; 3.91 (A part of an A_2B_2 spin system, *J*=9.7 Hz, 2H, H-1); 3.85 (B part of an A_2B_2 spin system, *J*=9.7 Hz, 2H, H-2); 3.30 (br s, 2H, H-6); 2.68 (t, *J*=5.8 Hz, 2H, H-8); 2.66-2.63 (overlapping m′s, 4H, N7-CH_2 and C-CH_2 (R^2)]; 2.58 (m, 2H, C-CH_2 (R^1)]; 2.48 (t, *J*=5.8 Hz, 2H, H-9). ^{13}C-NMR ($CDCl_3$): 161.5 (C-5); 152.9 (C-3a); 145.3 (C-9a); 101.9 (C-5a); 86.4 [C-1′ (R^1)]; 85.3 [C-1″ (R^2)]; 68.6 [two coalesced lines, η^5-C_5H_5 (R^1 and R^2)]; 68.23 and 68.18 [C-2′,5′ (R^1) and C-2″,5″ (R^2)]; 67.3 [two coalesced lines, C-3′,4′ (R^1) and C-3″,4″ (R^2)]; 59.4 (N7-CH_2); 50.6 (C-2); 49.5 (C-8); 49.0 (C-6); 46.8 (C-1); 43.0 (N4-CH_2); 27.7 [C-CH_2 (R^1)]; 26.7 (C-9); 26.6 [C-CH_2, (R^2)]. HRMS exact mass calculated for $C_{33}H_{36}Fe_2N_4O$: 616.1588; found for $[MH]^+$: 617.1642.

- **7-Ferrocenylmethyl-4-(2-methylbenzyl)-2,4,6,7,8,9-hexahydroimidazo[1,2-a]pyrido[3,4-e]pyrimidin-5(1*H*)-one** (**7db**): ^{1}H-NMR (DMSO-d_6): 7.09 [d, *J*=7.5 Hz, 1H, H-3″ (R^2)]; 7.06 [t, *J*=7.5 Hz, 1H, H-4″ (R^2)]; 7.02 [t, *J*=7.5 Hz, 1H, H-5″ (R^2)]; 6.88 [d, *J*=7.5 Hz, 1H, H-6″ (R^2)]; 7.32-7.29 [m, 4H, H-2′,3′5′,6′(R^1)]; 4.85 (s, 2H, N4-CH_2); 4.15 [br ~t, *J*~2 Hz, 2H, H-2′,5′ (R^1)]; 4.10 [s, 5H, η^5-C_5H_5 (R^1)]; 4.08 [t, *J*=2.1 Hz, 2H, H-3′,4′ (R^1)]; 3.87 (t, *J*=9.3 Hz, 2H, H-2); 3.64 (t, *J*=9.3 Hz, 2H, H-1); 3.44 (s, 2H, N7-CH_2); 2.99 (br s, 2H, H-6); 2.55 (t, *J*=5.5 Hz, 2H, H-8); 2.44 [t, *J*=5.5 Hz, H-9 (partly overlapped by the CD_2H signal of the solvent)]; 2.29 [s, 3H, CH_3 (R^2)]. ^{13}C-NMR (DMSO-d_6): 161.3 (C-5); 152.7 (C-3a); 147.5 (C-9a); 135.6 [C-1″ (R^2)]; 135.5 [C-2″ (R^2)]; 130.3 [C-3″ (R^2)]; 126.9 [C-4″ (R^2)]; 126.2 [C-5″ (R^2)]; 125.6 [C-6″ (R^2)]; 100.1 (C-5a); 83.6 [C-1′ (R^1)]; 70.2 [C-2′,5′ (R^1)]; 68.8 [η^5-C_5H_5 (R^1)]; 68.1 [C-3′,4′ (R^1)]; 57.1 (N7-CH_2); 50.5 (C-1); 48.6 (C-6); 48.4 (N-8); 46.8 (C-2); 42.7 (N4-CH_2); 26.3 (C-9); 19.2 [CH_3 (R^2)]. HRMS exact mass calculated for $C_{28}H_{30}FeN_4O$: 494.1769; found for $[MH]^+$: 495,1830.

- **7-Ferrocenylmethyl-4-(4-trifluoromethylbenzyl)-2,4,6,7,8,9-hexahydroimidazo[1,2-a]pyrido[3,4-e]pyrimidin-5(1*H*)-one** (**7dc**): ^{1}H-NMR ($CDCl_3$): 7.48 [s, 4H, H-2″,3″,5″,6″ (R^2)]; 5.05 (s, 2H, N4-CH_2); 4.16 [~t, *J*~2 Hz, 2H, H-2′,5′ (R^1)]; 4.10 [s, 5H, η^5-C_5H_5 (R^1)]; 4.08 [~t, *J*~2 Hz, 2H, H-3′,4′ (R^1)]; 3.83 (A part of an A_2B_2 spin system, *J*=9.7 Hz, 2H, H-2); 3.81 (B part of an A_2B_2 spin system, *J*=9.7 Hz, 2H, H-1); 3.50 (s, 2H, N7-CH_2); 3.20 (br s, 2H, H-6); 2.59 (t, *J*=5.8 Hz, 2H, H-8); 2.39 [t, *J*=5.8 Hz, H-9]. ^{13}C-NMR ($CDCl_3$): 161.4 (C-5); 153.0 (C-3a); 145.9 (C-9a); 140.9 [C-1″ (R^2)]; 129.5 [qa, *J*=32.2 Hz, C-4″ (R^2)]; 128.6 [C-2″,6″ (R^2)]; 125.3 [qa, *J*=3.8 Hz, C-3″,5″ (R^2)]; 124.7 [qa, *J*=272.5 Hz, CF_3 (R^2)]; 102.1 (C-5a); 82.2 [C-1′ (R^1)]; 70.2 [C-2′,5′ (R^1)]; 68.6 [η^5-C_5H_5 (R^1)]; 68.3 [C-3′,4′ (R^1)]; 57.5 (N7-CH_2); 50.6 (C-2); 48.7 (C-6); 47.8 (N-8); 46.9 (C-1); 44.9 (N4-CH_2); 26.7 (C-9). HRMS exact mass calculated for $C_{28}H_{27}F_3FeN_4O$: 548.1486; found for $[MH]^+$: 549.1543.

- **(±)-7-(2-Iodoferrocenylmethyl-4-(2-methylbenzyl)-2,4,6,7,8,9-hexahydroimidazo[1,2-a]pyrido[3,4-e]pyrimidin-5(1*H*)-one (7gb)**: ^{1}H-NMR (CDCl$_3$): 7.09-7.03 [overlapping m's, 3H, H-3″-5″ (R^2)]; 6.99 [m, 1H, H-6″ (R^2)]; 5.04 (A part of an AB spin system, *J*=15.5 Hz, 1H, N4-C$\underline{H}_A$H$_B$); 5.00 (B part of an AB spin system, *J*=15.5 Hz, 1H, N4-CH$_A\underline{H}_B$); 4.40 [dd, *J*=2.5 Hz and 1.4 Hz, 1H, H-3′ (R^1)]; 4.30 [dd, *J*=2.5 Hz and 1.4 Hz, 1H, H-5′ (R^1)]; 4.19 [t, *J*=2.5 Hz, 1H, H-4′ (R^1)]; 4.10 [s, 5H, η^5-C$_5\underline{H}_5$ (R^1)]; 3.86 (A part of an A$_2$B$_2$ spin system, *J*=9.7 Hz, 2H, H-2); 3.82 (B part of an A$_2$B$_2$ spin system, *J*=9.7 Hz, 2H, H-1); 3.58 (s, 2H, N7-C$\underline{H}_2$); 3.43 (A part of an AB spin system, *J*=14.6 Hz, 1H, H-6$_A$); 3.19 (B part of an AB spin system, *J*=14.6 Hz, 1H, H-6$_B$); 2.74-2.65 (m, 2H, H-8); 2.43 (~t, *J*~6 Hz, 2H, H-9); 2.36 [s, 3H, C$\underline{H}_3$ (R^2)]. ^{13}C-NMR (CDCl$_3$): 161.4 (C-5); 153.3 (C-3a); 145.6 (C-9a); 135.5 [C-2″ (R^2)]; 134.2 [C-1″ (R^2)]; 130.2 [C-3″ (R^2)]; 126.8 [C-4″ (R^2)]; 125.9 [C-5″ (R^2)]; 125.1 [C-6″ (R^2)]; 102.0 (C-5a); 84.2 [C-1′ (R^1)]; 75.0 [C-3′ (R^1)]; 71.7 [η^5-$\underline{C}_5$H$_5$ (R^1)]; 69.3 [C-4′ (R^1)]; 69.0 [C-5′ (R^1)]; 56.9 (N7-$\underline{C}$H$_2$); 50.6 (C-2); 49.1 (C-6); 48.0 (C-8); 46.9 (C-1); 46.3 [C-2′ (R^1)]; 44.7 (N4-$\underline{C}$H$_2$); 19.2 [$\underline{C}$H$_3$ (R^2)]. HRMS exact mass calculated for $C_{28}H_{29}FeIN_4O$: 620.0735; found for [MH]$^+$: 621.0795.

- **7-(4-Iodobenzyl)-4-(2-methylbenzyl)-2,4,6,7,8,9-hexahydroimidazo[1,2-a]pyrido[3,4-e]pyrimi-din-5(1*H*)-one (7hb)**: ^{1}H-NMR (CDCl$_3$): 7.61 [d, *J*=8.0 Hz, 2H, H-3′,5′ (R^1)]; 7.48 [s, 4H, H-2″,3″,5″,6″ (R^2)]; 5.05 (s, 2H, N4-C$\underline{H}_2$); 4.16 [~t, *J*~2 Hz, 2H, H-2′,5′ (R^1)]; 7.10-7.04 and 7.05 [m, 5H, H-3″-5″, (R^2) and *J*=8.0 Hz, H-2′,6′ (R^1), resp.]; 7.03 [m, 1H, H-6″ (R^2)]; 5.01 (s, N4-C$\underline{H}_2$); 3.88 (A part of an A$_2$B$_2$ spin system, *J*=9.5 Hz, 2H, H-2); 3.85 (B part of an A$_2$B$_2$ spin system, *J*=9.5 Hz, 2H, H-1); 3.57 (s, N7-C$\underline{H}_2$); 3.26 (br s, 2H, H-6); 2.63 (t, *J*=5.7 Hz, 2H, H-8); 2.47 (t, *J*=5.7 Hz, 2H, H-9); 2,37 [s, 3H, C$\underline{H}_3$ (R^2)]. ^{13}C-NMR (CDCl$_3$): 161.4 (C-5); 153.2 (C-3a); 145.6 (C-9a); 137.5 [two coalesced lines C-1′ and C-3′,5′ (R^1)]; 131.0 [C-2′,6′ (R^1)]; 130.2 [C-3″ (R^2)]; 126.8 [C-4″ (R^2)]; 125.9 [C-5″ (R^2)]; 125.2 [C-6″ (R^1)]; 101.9 (C-5a); 92.8 [C-4′ (R^1)]; 61.7 (N7-$\underline{C}$H$_2$); 50.6 (C-2); 49.5 (C-6); 48.2 (C-8); 47.0 (C-1); 43.2 (N4-$\underline{C}$H$_2$); 26.9 (C-9); 19.2 [$\underline{C}$H$_3$ (R^2)]. HRMS exact mass calculated for $C_{24}H_{25}IN_4O$: 512.1073; found for [MH]$^+$: 513.1140.

- **(±)-7-(2-Iodoferrocenylmethyl-4-(4-trifluoromethylbenzyl)-2,4,6,7,8,9-hexahydroimidazo[1,2-a]pyrido[3,4-e]pyrimidin-5(1*H*)-one (7gc)**: ^{1}H-NMR (CDCl$_3$): 7.50 [s, 4H, H-2″,3″,5″,6″ (R^2)]; 5.07 (A part of an AB spin system, *J*=15.2 Hz, 1H, N4-C$\underline{H}_A$H$_B$); 5.04 (B part of an AB spin system, *J*=15.2 Hz, 1H, N4-CH$_A\underline{H}_B$); 4.40 [dd, *J*=2.5 Hz and 1.4 Hz, 1H, H-3′ (R^1)]; 4.29 [dd, *J*=2.5 Hz and 1.4 Hz, 1H, H-5′ (R^1)]; 4.19 [t, *J*=2.5 Hz, 1H, H-4′ (R^1)]; 4.09 [s, 5H, η^5-C$_5\underline{H}_5$ (R^1)]; 3.84 (s, 4H, H-1 and H-2); 3.57 (s, 2H, N7-C$\underline{H}_2$); 3.41 (A part of an AB spin system, *J*=14.8 Hz, 1H, H-6$_A$); 3.17 (B part of an AB spin system, *J*=14.8 Hz, 1H, H-6$_B$); 2.72-2.62 (m, 2H, H-8); 2.40 (~t, *J*~6 Hz, 2H, H-9). ^{13}C-NMR (CDCl$_3$): 161.4 (C-5); 153.0 (C-3a); 145.9 (C-9a); 140.9 [C-1″ (R^2)]; 128.7 [C-2′,6′ (R^2)]; 129.5 [qa, *J*=32.2 Hz, C-4″(R^2)]; 128.7 [C-2″,6″ (R^2)]; 125.3 [qa, *J*=3.8 Hz, C-3″,5″ (R^2)]; 124.7 [qa, *J*=272.5 Hz, $\underline{C}$F$_3$ (R^2)]; 84.3 [C-1′ (R^1)]; 75.0 [C-3′ (R^1)]; 71.7 [η^5-$\underline{C}_5$H$_5$ (R^1)]; 69.3 [C-4′ (R^1)]; 69.0 [C-5′ (R^1)]; 57.0 (N7-$\underline{C}$H$_2$); 50.6 (C-2); 49.0 (C-6); 48.0 (C-8); 46.9 (C-1); 46.3 [C-2′ (R^1)]; 44.9 (N4-$\underline{C}$H$_2$); 26.8 (C-9). HRMS exact mass calculated for $C_{28}H_{26}F_3FeIN_4O$: 674.0453; found for [MH]$^+$: 674.0481.

- **(±)-7-(2-Iodoferrocenylmethyl-4-(4-iodobenzyl)-2,4,6,7,8,9-hexahydroimidazo[1,2-a]pyrido[3,4-e]pyrimidin-5(1*H*)-one (7gh)**: ^{1}H-NMR (CDCl$_3$): 7.56 [d, *J*=8.0 Hz, 2H, H-2″,6″ (R^2)]; 7.16 [d, *J*=8.0 Hz, 2H, H-3″,5″ (R^2)]; 4.95 (A part of an AB spin system, *J*=15.0 Hz, 1H, N4-C$\underline{H}_A$H$_B$); 4.93 (B part of an AB spin system, *J*=15.0 Hz, 1H, N4-CH$_A\underline{H}_B$); 4.40 [dd, *J*=2.5 Hz and 1.4 Hz, 1H, H-3′ (R^1)]; 4.28 [dd, *J*=2.5 Hz and 1.4 Hz, 1H, H-5′ (R^1)]; 4.19 [t, *J*=2.5 Hz, 1H, H-4′ (R^1)]; 4.09 [s, 5H, η^5-C$_5\underline{H}_5$ (R^1)]; 3.86-3.77 (m, 4H, H-1 and H-2); 3.56 (s, 2H, N7-C$\underline{H}_2$); 3.39 (A part of an AB spin system, *J*=14.6 Hz, 1H, H-6$_A$); 3.15 (B part of an AB spin system, *J*=14.6 Hz, 1H, H-6$_B$); 2.69-2.61 (m, 2H, H-8); 2.38 (~t, *J*~6 Hz, 2H, H-9). ^{13}C-NMR (CDCl$_3$): 161.4 (C-5); 152.9 (C-3a); 145.7 (C-9a); 137.4 [C-3″,5″ (R^2)]; 136.7 [C-1″ (R^2)]; 130.6 [C-2″,6″ (R^2)]; 102.0 (C-5a); 93.0 [C-4″ (R^2)]; 84.3 [C-1′ (R^1)]; 75.0 [C-3′ (R^1)]; 71.7 [η^5-$\underline{C}_5$H$_5$ (R^1)]; 69.3 [C-4′ (R^1)]; 69.0 [C-5′ (R^1)]; 56.9 (N7-$\underline{C}$H$_2$); 50.6 (C-2); 49.0 (C-6); 48.0 (C-8); 46.9 (C-1); 46.2 [C-2′ (R^1)]; 44.8 (N4-$\underline{C}$H$_2$); 26.7 (C-9). HRMS exact mass calculated for $C_{27}H_{26}FeI_2N_4O$: 731.9545; found for [MH]$^+$: 732.9600.

- **(±)-7-(2-Iodoferrocenylmethyl-4-(2-trifluoromethyl-4-fluorobenzyl)-2,4,6,7,8,9-hexahydroimidazo[1,2-a]pyrido[3,4-e]pyrimidin-5(1*H*)-one (7gi)**: ^{1}H-NMR (CDCl$_3$): 7.47 [t, *J*=7.6 Hz,

1H, H-5″ (R^2)]; 7.26 [br ~d, *J*~8 Hz, H, H-6″ (R^2)]; 7.23 [br ~d, *J*~11 Hz, H, H-2″ (R^2)]; 5.04 (A part of an AB spin system, *J*=15.0 Hz, 1H, N4-$C\underline{H}_AH_B$); 5.01 (B part of an AB spin system, *J*=15.0 Hz, 1H, N4-$CH_A\underline{H}_B$); 4.29 [dd, *J*=2.4 Hz and 1.3 Hz, 1H, H-3′ (R^1)]; 4.40 [dd, *J*=2.4 Hz and 1.3 Hz, 1H, H-5′ (R^1)]; 4.19 [t, *J*=2.4 Hz, 1H, H-4′ (R^1)]; 4.09 [s, 5H, η^5-$C_5\underline{H}_5$ (R^1)]; 3.84 (s, 4H, H-1 and H-2); 3.54 (s, 2H, N7-$C\underline{H}_2$); 3.40 (A part of an AB spin system, *J*=14.7 Hz, 1H, H-6_A); 3.16 (B part of an AB spin system, *J*=14.7 Hz, 1H, H-6_B); 2.71-2.63 (m, 2H, H-8); 2.40 (~t, *J*~6 Hz, 2H, H-9). ^{13}C-NMR ($CDCl_3$): 161.2 (C-5); 159.8 [dqa, *J*=256.2 Hz and 2.7 Hz, C-3″ (R^2)]; 152.9 (C-3a); 146.1 C-9a); 143.9 [d, *J*=7.0 Hz, C-1″ (R^2)]; 127.0 [qad, *J*=4.5 Hz and 1.6 Hz, C-5″ (R^2)]; 123.9 [d, *J*=3.1 Hz, C-6″ (R^2)]; 122.7 [qad, *J*=272.5 Hz and 1.4 Hz, $\underline{C}F_3$ (R^2)]; 117.2 [qad, *J*=33.0 Hz and 12.4 Hz, C-4″ (R^2)]; 116.6 [d, *J*=21.1 Hz, C-2″ (R^2)]; 84.1 [C-1′ (R^1)]; 75.0 [C-3′ (R^1)]; 71.7 [η^5-$\underline{C}_5H_5$ (R^1)]; 69.3 [C-4′ (R^1)]; 69.0 [C-5′ (R^1)]; 56.9 (N7-$\underline{C}H_2$); 50.6 (C-2); 48.8 (C-6); 47.9 (C-8); 46.9 (C-1); 46.2 [C-2′ (R^1)]; 44.6 (N4-$\underline{C}H_2$); 26.8 (C-9). HRMS exact mass calculated for $C_{28}H_{25}F_4FeIN_4O$: 692.0359; found for $[MH]^+$: 693.0412.

- **(±)-7-Benzyl-4-(2-iodoferrocenylmethyl)-2,4,6,7,8,9-hexahydroimidazo[1,2-a]pyrido[3,4-e]pyrimidin-5(1*H*)-one** (**7ag**): ^{1}H-NMR ($CDCl_3$): 7.32-7.20 [overlapping m′s, 5H, H-2′-6′ (R^1)]; 6.99 [m, 1H, H-6″ (R^2)]; 5.01 (A part of an AX spin system, *J*=14.4 Hz, 1H, N4-$C\underline{H}_AH_X$); 4.76 (X part of an AX spin system, *J*=14.4 Hz, 1H, N4-$CH_A\underline{H}_X$); 4.43 [dd, *J*=2.5 Hz and 1.4 Hz, 1H, H-3″ (R^2)]; 4.35 [dd, *J*=2.5 Hz and 1.4 Hz, 1H, H-5″ (R^2)]; 4.14 [s, 5H, η^5-$C_5\underline{H}_5$ (R^2)]; 4.09 [t, *J*=2.5 Hz, 1H, H-4″ (R^2)]; 3.88 (A part of an A_2B_2 spin system, *J*=9.6 Hz, 2H, H-1); 3.83 (B part of an A_2B_2 spin system, *J*=9.6 Hz, 2H, H-2); 3.67 (s, 2H, N7-$C\underline{H}_2$); 3.64 (A part of an AB spin system, *J*=14.3 Hz, 1H, H-6_A); 3.62 (B part of an AB spin system, *J*=14.3 Hz, 1H, H-6_B); 2.61 (t, *J*=5.8 Hz, 2H, H-8); 2.41 (t, *J*=5.8 Hz, 2H, H-9). ^{13}C-NMR ($CDCl_3$): 161.2 (C-5); 152.9 (C-3a); 145.4 (C-9a); 137.8 [C-1′ (R^1)]; 129.1 [C-2′,6′ (R^1)]; 128.4 [C-3′,5′ (R^1)]; 127.3 [C-4′ (R^1)]; 102.1 (C-5a); 85.5 [C-1″ (R^2)]; 73.9 [C-3″ (R^2)]; 71.9 [η^5-$\underline{C}_5H_5$ (R^2)]; 69.1 [C-5″ (R^2)]; 68.2 [C-4″ (R^2)]; 62.3 (N7-$\underline{C}H_2$); 50.4 (C-2); 49.7 (C-6); 48.3 (C-8); 46.2 (C-1); 43.4 [C-2″ (R^2)]; 41.9 (N4-$\underline{C}H_2$); 26.8 (C-9). HRMS exact mass calculated for $C_{27}H_{27}FeIN_4O$: 606.0579; found for $[MH]^+$: 607.0636.

- **(±)-7-(1-ferrocenylpropan-2-yl)-4-(2-methylbenzyl)-2,4,6,7,8,9-hexahydroimidazo[1,2-a]pyrido[3,4-e]pyrimidin-5(1*H*)-one** (**7fb**): ^{1}H-NMR ($CDCl_3$): 7.11-7.03 [overlapping m′s, 4H, H-3″-6″ (R^2)]; 5.04 (s, 2H, N4-$C\underline{H}_2$); 4.06 [s, 5H, η^5-$C_5\underline{H}_5$ (R^1)]; 4.03 and 4.01 (2xbr s, 2x1H, H-2′ and H-5′ (R^1)]; 4.05 [br s, 2H, H-3′,4′ (R^1)]; 3.89 (A part of an A_2B_2 spin system, *J*=9.6 Hz, 2H, H-1); 3.85 (B part of an A_2B_2 spin system, *J*=9.6 Hz, 2H, H-2); 3.40 (A part of an AB spin system, *J*=14.9 Hz, 1H, H-6_A); 3.36 (B part of an AB spin system, *J*=14.9 Hz, 1H, H-6_B); 3.31 [dd, *J*=14.3 Hz and 10.4 Hz, 1H, C-$C\underline{H}_AH_X$ (R^1)]; 2.75-2.68 (overlapping m′s, 4H, H-8, N7-C$\underline{H}$ and C-$CH_A\underline{H}_X$ (R^1)]; 2.51-2.46 (m, 2H, H-9); 2.39 [s, 3H, $C\underline{H}_3$ (R^2)]; 0.95 (d, *J*=6.4 Hz, 3H, $C\underline{H}_3$ (R^1)]. ^{13}C-NMR ($CDCl_3$): 161.8 (C-5); 153.4 (C-3a); 145.9 (C-9a); 135.6 [C-1″ (R^2)]; 134.3 [C-2″ (R^2)]; 130.2 [C-3″ (R^2)]; 126.9 [C-5″ (R^2)]; 126.0 [C-4″ (R^2)]; 125.4 [C-6″ (R^2)]; 102.5 (C-5a); 86.3 [C-1′ (R^1)]; 69.7 [C-3′,4′ (R^1)]; 68.6 [η^5-$\underline{C}_5H_5$ (R^1)]; 67.6 and 67.2 [C-2′ and C-5′ (R^1)]; 61.4 (N7-$\underline{C}$H); 50.6 (C-2); 47.0 (C-1); 46.3 (C-6); 44.8 (C-8); 43.2 (N4-$\underline{C}H_2$); 27.5 (C-9); 19.3 [$\underline{C}H_3$ (R^2)]; 14.2 [$\underline{C}H_3$ (R^1)]. HRMS exact mass calculated for $C_{30}H_{34}FeN_4O$: 522.2082; found for $[MH]^+$: 523.2146.

- **4-(2-Ferrocenylethyl)-7-ferrocenylmethyl-2,4,6,7,8,9-hexahydroimidazo[1,2-a]pyrido[3,4-e]pyrimidin-5(1*H*)-one** (**7de**): ^{1}H-NMR ($CDCl_3$): 4.18 [t, *J*=2.0 Hz, 2H, H-2′,5′ (R^1)]; 4.12 [s, 5H, η^5-$C_5\underline{H}_5$ (R^1)]; 4.10 [coalesced s′s, 9H, H-3′,4′ (R^1), H-2″,5″ (R^2) and η^5-$C_5\underline{H}_5$ (R^2)]; 4.02-3.98 (overlapping m′s, 4H, N4-$C\underline{H}_2$ and H-3″,4″ (R^2)]; 3.86 (t, *J*=9.7 Hz, 2H, H-1); 3.77 (t, *J*=9.7 Hz, 2H, H-2); 3.51 (s, N7-$C\underline{H}_2$); 3.22 (br s, 2H (H-6); 2.61-2.58 [overlapping m′s, H-8 and C-$C\underline{H}_2$ (R^2)]; 2.29 (t, *J*=5.8 Hz, H-9). ^{13}C-NMR ($CDCl_3$): 161.4 (C-5); 152.9 (C-3a); 145.2 (C-9a); 102.1 (C-5a); 85.2 [C-1″ (R^2)]; 82.2 [C-1′ (R^1)]; 70.2 [C-2′,5′ (R^1)]; 68.6 [two coalesced lines, η^5-$\underline{C}_5H_5$ (R^1 and R^2)]; 68.2 [C-2″,5″ (R^2)]; 68.1 [C-3′,4′ (R^1)]; 67.3 [C-3″,4″ (R^2)]; 57.6 (N7-$\underline{C}H_2$); 50.5 (C-2); 48.7 (C-6); 47.9 (C-8); 46.7 (C -1); 43.0 (N4-$\underline{C}H_2$); 26.7 [two coalesced lines, C-9 and C-$\underline{C}H_2$ (R^2)]. HRMS exact mass calculated for $C_{32}H_{34}Fe_2N_4O$: 602.1431; found for $[MH]^+$: 603.1483.

- **7-(2-Ferrocenylethyl)-4-ferrocenylmethyl-2,4,6,7,8,9-hexahydroimidazo[1,2-a]pyrido[3,4-e]pyrimidin-5(1*H*)-one** (**7ed**): ^{1}H-NMR (DMSO-d_6): 4.63 (s, 2H, N4-$C\underline{H}_2$); 4.27 [br s, 2H, H-2″,5″ (R^2)]; 4.13 [s, 5H, η^5-$C_5\underline{H}_5$ (R^2)]; 4.07 [two coalesced s′s, 7H, H-2′,5′ and η^5-$C_5\underline{H}_5$ (R^1)]; 4.00 [br ~t, *J*~2 Hz, 2H, H-3″,4″ (R^2)]; 3.98 [br ~t, *J*~2 Hz, 2H, H-3′,4′ (R^1)]; 3.84 (t, *J*=9.3 Hz, 2H, H-2); 3.69 (t, *J*=9.3 Hz, 2H, H-1);

3.02 (br s, 2H, H-6); 2.55-2.51 (overlapping m's, 4H, H-8 and N7-C<u>H</u>$_2$); 2.41-2.38 [overlapping m's, 4H, H-9 and C-C<u>H</u>$_2$ (R^1)]. ^{13}C-NMR (DMSO-d_6): 160.9 (C-5); 152.4 (C-3a); 147.2 (C-9a); 100.1 (C-5a); 87.2 [C-1′ (R^1)]; 83.3 [C-1″ (R^2)]; 70.5 [C-2″,5″ (R^2)]; 68.8 [two coalesced lines, η^5-<u>C</u>$_5$H$_5$ (R^1) and η^5-<u>C</u>$_5$H$_5$ (R^2)]; 68.1 [C-2′,5′ (R^1)]; 67.9 [C-3″,4″ (R^2)]; 67.4 [C-3″,4″ (R^1)]; 58.7 (N7-<u>C</u>H$_2$); 50.7 (C-2); 49.3 (C-6); 49.0 (C-9); 46.7 (C-1); 40.8 (N4-<u>C</u>H$_2$); 27.2 [C-<u>C</u>H$_2$ (R^1)]; 26.2 (C-9). HRMS exact mass calculated for $C_{32}H_{34}Fe_2N_4O$: 602.1431; found for [MH]$^+$: 603.1488.

- **4,7-Bis-(4-iodophenylmethyl)-2,4,6,7,8,9-hexahydroimidazo[1,2-a]pyrido[3,4-e]pyrimidin-5(1*H*)-one (7hh)**: ^{1}H-NMR (CDCl$_3$): 7.61 (d, *J*=8.2 Hz, 2H, H-3′,5′ (R^1)]; 7.57 (d, *J*=8.2 Hz, 2H, H-3″,5″ (R^2)]; 7.12 [d, *J*=8.2 Hz, 2H, H-2″,6″ (R^2)]; 7.04 (d, *J*=8.2 Hz, 2H, H-2′,6′ (R^1)]; 4.94 (s, 2H, N4-C<u>H</u>$_2$); 3.86 (A part of an A$_2$B$_2$ spin system, *J*=9.5 Hz, 2H, H-2); 3.84 (B part of an A$_2$B$_2$ spin system, *J*=9.5 Hz, 2H, H-1); 3.62 (s, N7-C<u>H</u>$_2$); 3.22 (br s, 2H, H-6); 2.60 (t, *J*=5.7 Hz, 2H, H-8); 2.41 (t, *J*=5.7 Hz, 2H, H-9). ^{13}C-NMR (CDCl$_3$): 161.3 (C-5); 152.9 (C-3a); 145.6 (C-9a); 137.52 [C-3′,5′ (R^1)]; 137.49 [C-1′ (R^1)]; 137.4 [C-3″,5″ (R^2)]; 136.6 [C-1″ (R^2)]; 131.0 [C-2′,6′ (R^1)]; 130.8 [C-2″,6″ (R^2)]; 101.8 (C-5a); 93.1 [C-4″ (R^2)]; 92.8 [C-4′ (R^1)]; 61.6 (N7-<u>C</u>H$_2$); 50.5 (C-2); 49.4 (C-6); 48.2 (C-8); 46.9 (C-1); 44.9 (N4-<u>C</u>H$_2$); 26.8 (C-9). HRMS exact mass calculated for $C_{23}H_{22}I_2N_4O$: 623.9883; found for [MH]$^+$: 624.9941.

3. *Model cells and culturing*

The five investigated tumor cell lines – PANC-1 (human pancreatic carcinoma of ductal origin), COLO 205 (human colon adenocarcinoma), EBC-1 (human lung squamous cell carcinoma), A2058 (human malignant melanoma with high invasiveness), HT-29 (human well-differentiated colon adenocarcinoma) were obtained from European Collection of Authenticated Cell Cultures (ECACC, Salisbury, UK).

Dulbecco's Modified Eagle Medium (DMEM, Lonza, Basel, Switzerland) supplemented with 10% foetal bovine serum (FBS, Gibco®/Invitrogen Corporation, New York, NY, USA), l-glutamine (2 mmol/L) (Lonza, Basel, Switzerland) and 100 μg/mL penicillin/streptomycin (Gibco®/Invitrogen Corporation, New York, NY, USA). (Gibco®/Invitrogen Corporation, New York, NY, USA) was used for culturing PANC-1 cells. This medium was further supplemented with D-glucose at a final concentration of 4500 mg/L (Sigma-Aldrich, St. Louis, MO, USA) in case of COLO 205 cell line, while 1% non-essential amino acids (NEAA, Gibco®/Invitrogen Corporation, New York, NY, USA), 1 mM sodium pyruvate (Sigma-Aldrich, St. Louis, MO, USA) was added the above-mentioned medium to culture the EBC-1 cells.

For the long term cytotoxicity studies the cultures of A2058 and HT-29 were maintained in RPMI 1640 (Lonza, Basel, Switzerland) containing 10 % FBS (Gibco®/Invitrogen Corporation, New York, NY, USA), 2 mmol/L L-glutamine (Lonza, Basel, Switzerland) and 100 μg/ml penicillin/streptomycin (Gibco®/Invitrogen Corporation, New York, NY, USA). All cell lines were maintained in plastic culture dishes (Sigma-Aldrich, St. Louis, MO, USA or Eppendorf AG, Hamburg, Germany) at 37 °C in a humidified 5% CO_2 atmosphere.

3.1. *Proliferation and cytotoxicity assays*

3.1.1. *Impedimetry*

The antiproliferative/cytotoxic effect of the novel impiridones on PANC-1 model cells was determined by the xCELLigence SP System (ACEA Biosciences, San Diego, CA, USA). The The principle of impedimetric measurement was previously described in detail [1]. In brief, the system is dedicated to

measure the kinetics and strength of cell attachment and proliferation by monitoring electrical impedance in real-time across gold microelectrodes fabricated to the bottom of a so-called E-plate. The recorded cell index (CI) values are calculated from the difference between impedance values at a given time point and at t = 0 h divided by a constant depending on the frequency of AC current.

For the experiments, the novel ONC201 hybrids were solved in DMSO to prepare the stock solution. The serial dilutions ranged from 2.5 x 10^{-5} to 10^{-7} M was made from the stock solution in supplemented DMEM medium.

To gain a background curve of constant CI value, 100 μL of pure cell culture medium was added to each well and the CI was recorded for 30-60 min. In the following step, 10^4 cells/well were loaded on the E-plate and monitored until the CI curves reaching a plateau phase showing a balanced state of PANC-1 cells (for roughly 24 h). The test compounds were added to the cells at this plateau phase, then the changes in CI were monitored for at least 72 hours at 10 kHz. The wells loaded with an adequate volume ratio of DMSO served as a control. The time course CI values of each concentration of the identical compounds were normalized to that of the DMSO control. Each measurement was carried out in triplicates. The normalized CI values obtained at 24, 48 and 72 h for each concentration were used the calculation of IC_{50} values by fitting sigmoidal dose-response curve with the nonlinear regression function of OriginPro 8 (OriginLab Corporation, Northampton, MA, USA).

3.1.2. Colorimetric assay

In the case of COLO 205, A2058 and EBC-1 cell lines a colorimetric assay (alamarBlue- or MTT-test) was used to determine the antiproliferative/cytotoxic effects of the ONC201 derivatives. These colorimetric assays were chosen because for these cell lines no stable plateau phase (A2058) or only a weak/negligible adhesion (COLO 205 and EBC-1) was to be able to detect with the xCELLigence System.

Prior to the treatment, COLO 205, EBC-1 and A2058 cells were seeded on 96-well plates (Sarstedt AG, Nümbrecht, Germany) in 10^4 cells/well concentration. After culturing the cells for 24 h, they were treated with the test compounds using the same concentration range described above. To determine the viability of the cells incubated for 24, 48 and 72 h, the alamarBlue (0.15 mg/mL, Sigma-Aldrich, St. Louis, MO, USA) reagent solved in PBS (phosphate-buffered saline, pH = 7.2) was added to the wells. After overnight incubation with the alamarBlue, the fluorescence intensity of the samples was read with LS-50B Luminescence Spectrometer (Perkin Elmer Ltd., Buckinghamshire United Kingdom) by using λ = 560 nm for the excitation and λ = 590 nm for the detection of emitted light. In this experiment, there were 3 parallels for each concentration. The control wells contained adequate volume ratio of DMSO. In order to calculate the IC_{50} values, sigmoidal dose-response curves were fitted to the fluorescence intensity data normalized to the DMSO control with the nonlinear regression function of OriginPro 8 (OriginLab Corporation, Northampton, MA, USA).

2.1.2. Statistical evaluation of data

Evaluation of the results was performed using RTCA 2.0 (ACEA Biosciences, San Diego, CA, USA), MS Excel, OriginPro 8 (OriginLab Corporation, Northampton, MA, USA) software. Data obtained

from each experiment represent mathematical averages. The standard deviations of IC_{50} parameters were also obtained with the sigmoidal curve fitting.

4. *Short term cytotoxicity studies using MTT assay*

For the short term cytotoxicity studies the HT-29- and A2058 cells were cultured in RPMI-1640 medium supplemented with 10% FCS (fetal calf serum, Sigma Ltd.), 2mM L-glutamine, and 160 mg/mL gentamicin. Cell cultures were maintained at 37 °C in a humidified atmosphere with 5% CO_2. The cells were grown to confluency and were distributed into 96-well plate with initial cell number of $5.0x10^3$ per well. After 24 h incubation at 37 °C, the cells were treated with the compounds in 200 µL final volume containing 1.0 v/v% DMSO. The cells were incubated with the compounds at 10^{-4}-10^2 µM concentration range for 1 h. Control cells were treated with serum free medium (RPMI-1640) only or with DMSO (c = 1.0 v/v %) at 37 °C for 1 h. After incubation the cells were washed twice with serum free (RPMI-1640) medium. To determine the *in vitro* cytostatic effect, the cells were cultured for a further 72 h in 10% serum containing medium. MTT-solution (45 mL, 2 mg/mL, final concentration: 0.37 mg/mL) was added to each well. The respiratory chain [2] and other electron transport systems [3] reduce MTT and thereby form non-water-soluble violet formazane crystals within the cell [4]. The amount of these crystals can be determined spectrophotometrically and serves as an estimate for the number of mitochondria and hence the number of living cells in the well [5]. After 4 h of incubation the cells were centrifuged for 5 min (900 g) and the supernatant was removed. The obtained formazane crystals were dissolved in DMSO (100 mL) and optical density (OD) of the samples was measured at λ = 540 and 620 nm, respectively, using ELISA Reader (iEMS Reader, Labsystems, Finland). OD_{620} values was substracted from OD_{540} values. The percent of cytostasis was calculated by using the following equation:

$$\text{Cytostatic effect (\%)} = [1-(OD_{treated}/OD_{control})]x100$$

Values $OD_{treated}$ and $OD_{control}$ correspond to the optical densities of the treated and the control cells, respectively. In each case two independent experiments were carried out with 4 parallel measurements. The 50% inhibitory concentration (IC_{50}) values were determined from the dose-response curves. The curves were defined using Microcal TM Origin1 (version 7.5) software: cytostasis was plotted as a function of concentration, fitted to a sigmoidal curve, and based on this curve, the half maximal inhibitory concentration (IC_{50}) value was determined. IC_{50} represents the concentration of a compound that is required for 50% inhibition *in vitro* and expressed in micromolar units.

References

1. Lajkó, E.; Szabó, I.; Andódy, K.; Pungor, A.; Mező, G.; Kőhidai, L. Investigation on chemotactic drug targeting (chemotaxis and adhesion) inducer effect of GnRH-III derivatives in Tetrahymena and human leukemia cell line. *J. Pept. Sci.* **2013**, *19*, 46–58. https://doi.org/10.1002/psc.2472.

2. a) Slater, T.F.; Sawyer, B.; Strauli, U. Studies on succinate-tetrazolium reductase systems: III. Points of coupling of four different tetrazolium salts III. Points of coupling of four different tetrazolium salts. *Biochim Biophys Acta*. **1963**, *77*, 383-393. https://doi.org/10.1016/0006-3002(63)90513-4.; (b) Mosmann, T.J. Rapid colorimetric assay for cellular growth and survival: application to proliferation and cytotoxicity assays. *Immunol. Methods*. **1983**, *65*, 55-63. https://doi.org/10.1016/0022-1759(83)90303-4

3. Liu, Y.B.; Peterson, D.A.; Kimura, H.; Schubert, D.J. Mechanism of Cellular 3-(4,5-Dimethylthiazol-2-yl)-2,5-Diphenyltetrazolium Bromide (MTT) Reduction. *Neurochem*. **1997**, *69*, 581-593. DOI: 10.1046/j.1471-4159.1997.69020581.x

4. Altman, F.P. Tetrazolium salts and formazans**.** *Prog. Hystochem. Cytochem*. **1976**, *9*, 1-56. PubMed ID: 792958

5. Denizot, F.; Lang, R. Rapid colorimetric assay for cell growth and survival. Modifications to the tetrazolium dye procedure giving improved sensitivity and reliability. *J. Immunol Methods*. **1986**, *89*, 271-277. PubMed ID: 3486233.
